# A novel plasmid-based co-tethered transcription platform for high yield, high purity mRNA synthesis

**DOI:** 10.1093/nar/gkaf1355

**Published:** 2025-12-29

**Authors:** Purnima Mala, Ruptanu Banerjee, Amin Abek, James Forster III, Aniruddha Pinjari, Ashish A Kulkarni, Craig T Martin

**Affiliations:** Department of Chemistry, University of Massachusetts Amherst, 710 N Pleasant St, Amherst, MA 01003, United States; Department of Chemistry, University of Massachusetts Amherst, 710 N Pleasant St, Amherst, MA 01003, United States; Department of Chemistry, University of Massachusetts Amherst, 710 N Pleasant St, Amherst, MA 01003, United States; Department of Chemical Biomolecular Engineering, University of Massachusetts Amherst, 686 North Pleasant StreetAmherst, MA 01003, United States; Department of Chemical Biomolecular Engineering, University of Massachusetts Amherst, 686 North Pleasant StreetAmherst, MA 01003, United States; Department of Chemical Biomolecular Engineering, University of Massachusetts Amherst, 686 North Pleasant StreetAmherst, MA 01003, United States; Department of Chemistry, University of Massachusetts Amherst, 710 N Pleasant St, Amherst, MA 01003, United States

## Abstract

This work aims to improve RNA synthesis and manufacturing, exemplified by T7 RNA polymerase-driven *in vitro* transcription. We developed a novel, plasmid-compatible co-tethering strategy that functionally couples RNA polymerase to its promoter DNA immobilized on a solid matrix. As demonstrated recently, co-tethering enhances promoter binding, increases RNA yield, and suppresses RNA re-binding, especially under high-salt conditions, thereby reducing double-stranded RNA by-products. The system leverages asymmetric end-labeling of linearized plasmid DNA using a simple “Klenow fill-in” reaction with modified nucleotides, enabling stable attachment of DNA to both RNA polymerase and solid support (magnetic beads). The immobilized co-tethered polymerase–DNA complex supports efficient transcription initiation in high-salt environments (which further reduces RNA re-binding), yielding RNA of high purity. Co-tethered complex remains functionally stable over extended storage and multiple transcription cycles (10-20 rounds), re-using the enzyme–DNA catalyst. Transcripts of lengths (0.8, 5.6, and 8.6 kb) are efficiently produced. Highly sensitive *in vitro* assays with immune cells confirm low immunogenicity and strong translational output, while *in vivo* validation using a novel Matrigel-plugged mouse model demonstrates robust expression and safety. With a simple modification to the DNA template, the reusable, co-tethered enzyme–DNA catalytic complex streamlines mRNA manufacturing by producing RNA of higher purity from the outset.

## Graphical Abstract

**Graphical Abstract:**
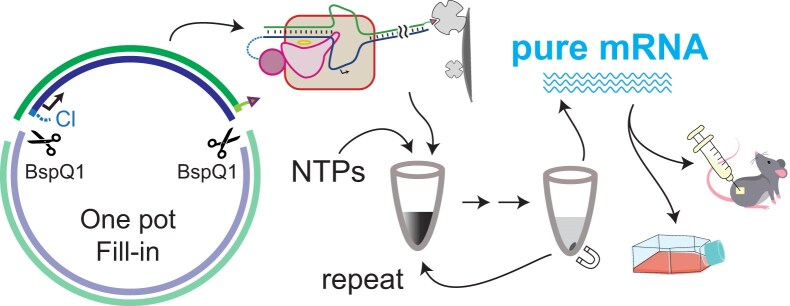

## Introduction

T7 RNA polymerase is a highly efficient single-subunit phage RNA polymerase that synthesizes larger RNA during run-off transcription [1, 2]. RNA synthesis occurs primarily when RNA polymerase binds to RNA-encoding DNA dissolved in solution. Some methods involve immobilizing RNA-encoding DNA on a solid matrix while keeping the RNA polymerase enzyme in solution [3]. Both approaches face the challenge of accumulating free RNA that competes with promoter DNA for binding to the free RNA polymerase. The re-binding of free RNA polymerase to RNA can result in the synthesis of partial double-stranded RNA (dsRNA) impurities [4–6]. Functionally co-tethering RNA polymerase to RNA-encoding DNA enhances promoter rebinding and enables transcription initiation at salt concentrations that reduce the generation of dsRNA impurities [7, 8]. The co-tethering method requires the addition of chemical “handles” in the DNA to facilitate tethering to proteins and a solid surface. In initial studies, these handles were incorporated into PCR (polymerase chain reaction)-generated DNA by including them in the PCR primers. In RNA synthesis and manufacturing, linearized plasmid DNA is commonly utilized as the RNA-encoding DNA and is sometimes preferred over DNA produced by amplification methods like PCR [9, 10]. Plasmid DNA generated in bacteria is more cost-effective and promises higher fidelity compared to multiple rounds of PCR amplification, which can introduce mutations into the DNA [11]. PCR amplification becomes increasingly challenging with longer RNA-encoding DNA and is less commonly used as lengths increase. Segmented poly(A) tails are now routinely incorporated [12] into plasmid-based DNA templates. Strategic methods (e.g. capping efficiency optimization, adjusting the unmodified/modified nucleotide ratio, sequence-structure optimization, and enhancements to downstream purification) have been developed to improve the quality, stability, and yield of mRNA.

**Figure 1.**
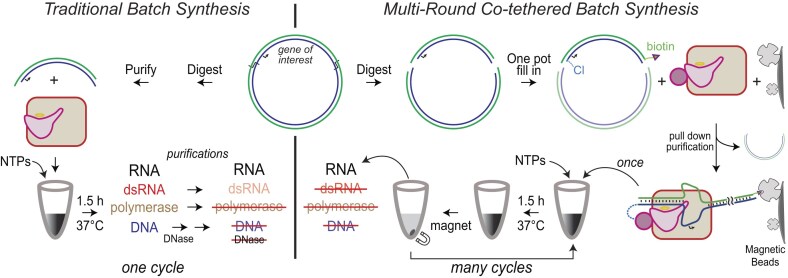
Comparison of traditional batch synthesis versus co-tethered plasmid-based transcription systems. In conventional transcription, plasmid DNA is digested and purified to obtain the functional DNA template. A single round of transcription ends with multiple purification steps. In co-tethered transcription, digested plasmid DNA is adapted in a one-pot fill-in reaction and then bound to RNA polymerase and to magnetic beads. A magnetic bead pulldown readies DNA and polymerase for transcription. At the end of RNA synthesis, bead-bound polymerase and DNA are pulled down, yielding pure RNA. The co-tethered system is then ready for another round of transcription. Anchoring RNA polymerase at the promoter (allowing high salt) eliminates dsRNA production from the outset.

Asymmetric labeling of DNA with recessed 3′ termini can be achieved via DNA polymerase (Klenow) fill-in reaction(s) [13]. In this study, we explored “fill-in” to achieve one-pot dual labeling of linearized plasmid DNA for immobilization and RNA flow manufacturing. By simply designing sequences outside the RNA-encoding region, as shown in Fig. 1, we achieve a one-pot fill-in reaction that yields asymmetrically labeled promoter-containing DNA without labeling the remaining plasmid vector. The incorporated reactive handles enable tethering the enzyme near the upstream promoter and facilitate attachment of the DNA to the solid matrix. We demonstrate transcription from co-tethered linear plasmid DNA at high salt (0.3 M) to largely eliminate polymerase rebinding to RNA, thereby reducing dsRNA by-products.

The power of the co-tethering approach lies in the ability to decorate the DNA with various labels according to the needs of the experiment. Our decorated plasmid DNA template contains an alkyl halide linker upstream and a biotin linker downstream for constructing a co-tethered plasmid DNA–T7 RNA polymerase complex to allow longer polynucleotide modification, such as plasmid DNA, with acceptable tags, eliminating chemical synthesis or error-prone polymerase chain reaction-based DNA modification. We successfully showed that our one-pot plasmid DNA labeling approach worked on RNA-encoding plasmid DNA up to 8647 base pairs. Modified nucleotide incorporation in the plasmid DNA terminus is specific and works at more than 95% modification efficiency.

## Materials and methods

All institutional and national guidelines for the care and use of laboratory animals were followed and approved by the University of Massachusetts Amherst Institutional Use and Care of Animals Committee (Approval # 5870).

### RNA-encoding plasmid DNA sequence

DNA encoding a nanoluciferase (NLuc) gene was prepared by Aldevron. Plasmids pCMV-T7-SpCas9-P2A-EGFP (Addgene, 139987) and pcDNA-ELYS–Emerin–EGFP-polyA (ELYS—dual nucleoporin/kinetochore protein required for nuclear pore assembly, Emerin—inner nuclear membrane protein) (Addgene, 59745) used in this study were a gift from Benjamin Kleinstiver [14] and Yi Zhang [15], respectively. These plasmids were edited to incorporate mRNA elements, including 5′ and 3′ untranslated regions (UTRs) and poly(A) tails. All three were designed with BspQ1 sites only at the upstream and downstream ends of the genes, which, after linearization, can encode 0.8 kb (NLuc), 5.6 kb (Cas9–EGFP), and 8.6 kb (ELYS–Emerin–EGFP) mRNA. Plasmid Nanoluciferase and pcDNA-ELYS–Emerin–EGFP-polyA were also designed/edited to include asymmetric overhangs as described below. The sequences of the DNA templates are listed in the supplementary data. Note that the plasmid pCMV-T7-SpCas9-P2A-EGFP does not encode 5′ or 3′ UTRs and does not encode a poly(A) tail.

### Preparation of Cl-alkyl-dUTP

As illustrated in Supplementary Fig. S1, amino-allyl-dUTP was modified in-house to Cl-alkyl-amino-allyl-dUTP (Cl-alkyl-dUTP). Briefly, 1 mM amino-allyl-dUTP (Jena Biosciences, NU-803S) was mixed with 6 mM HaloTag^®^ ligand succinimidyl ester (O4) (Promega, P6751) in 1× phosphate buffer saline (PBS), pH 7.4, and incubated at room temperature for 4 h. Since results (see Supplementary Fig. S1A) showed >95% conversion, the product was used directly in labeling DNA (Cl-alkyl-dUTP was stored at −80°C and remains stable for at least 3 months). The resulting Cl-alkyl-dUTP is accepted as a substrate in the “Klenow fill-in” reaction by DNA Polymerase I, Large (Klenow) Fragment (New England Biolabs, M0210S).

### Plasmid DNA digestion with BspQ1

Plasmid DNA (100 nM) was linearized with BspQ1 (New England Biolabs, 0712S) at an incubation temperature of 50°C for 1 h in NEB 2.0 buffer. BspQ1 was then heat-inactivated at 80°C for 20 min. The linearized plasmid DNA template was then used directly for DNA Polymerase I, Large (Klenow) Fragment-based “Klenow fill-in” reaction to incorporate a specific label at both 3′ termini (upstream and downstream). Alternatively, BspQ1-HF^®^ (New England Biolabs, R3712S) is used in rCutSmart buffer for plasmid DNA digestion and Klenow fill-in reaction with great success.

### One-pot plasmid DNA labeling

To incorporate chemical handles, 1 μg of 87, 806, 5659, or 8647 bp BspQI-cleaved DNA was incubated with 33 μM Cl-alkyl-dUTP (see above), 33 µM biotin-16-dCTP (Jena Biosciences, NU-809-BIO16-S), and 1 U Klenow polymerase in the supplied NEB 2.0 buffer. Reactions were incubated at 25°C for 15 min (87 bp DNA) or 2–4 h (806, 5659, and 8647 bp DNA). Labeled DNA was purified with a DNA cleanup kit or ethanol precipitation.

### DNA immobilization to hydrophilic streptavidin magnetic beads

To immobilize DNA to magnetic beads, 1 µg cleaned-up labeled DNA (806 bp) was added to 50 μg of hydrophilic streptavidin-coated magnetic beads (New England Biolabs, S1421S) in 20 μl of 1× PBS, pH 7.4, for 60 min at room temperature. For larger DNA (5659 and 8647 bp), 100 μg beads were used (Supplementary Fig. S2, lane 5). The supernatant was removed, and the beads were washed three times with 100 μl of streptavidin wash buffer [20 mM Tris-Cl, pH 7.5, 1 mM ethylenediaminetetraacetic acid (EDTA), 0.5 M NaCl, 0.1% Tween-20]. To analyze the DNA to bead binding efficiency, the supernatant is run in a 2% agarose gel and is found to bind (>95%) completely (Supplementary Fig. S2).

### Stability of modified DNA from exonuclease degradation

The DNA, being modified at the 3′ end, is hypothesized to be protected from 3′ exonuclease-mediated degradation, rendering tolerability of exonuclease contaminations (if any) in subsequent reactions. We tested Exonuclease III (3′–5′), DNase I (endonuclease), and T5 exonuclease (5′–3′) with the labeled Cas9–EGFP DNA. Briefly, 1 µg of labeled Cas9–EGFP DNA was incubated with Exonuclease III, DNase I, and T5 exonuclease, separately with NEBuffer 1, DNase I buffer, and NEBuffer 4, respectively, for 30 min at 37°C. A 2% agarose gel electrophoresis was performed to visualize the nuclease-mediated degradation (Supplementary Fig. S3) with linearized plasmid DNA as a negative control.

### Covalent coupling of DNA to HaloTag-T7 RNA polymerase

Finally, the bead-bound DNA template was suspended in 20 µl of 1× PBS (pH 7.4) with 0.1 µM HaloTag-T7 RNA polymerase [7] and incubated at room temperature for 60 min. The supernatant was removed, and the bead-bound enzyme–DNA complex was washed three times with 100 µl streptavidin wash buffer (20 mM Tris-Cl, pH 7.5, 1 mM EDTA, 0.5 M NaCl, 0.1% Tween-20) using a magnetic rack (New England Biolabs, S1506S) to exchange buffers.

### Traditional batch transcription

DNA and T7 RNA polymerase were added with 1× transcription buffer [40 mM magnesium acetate, 30 mM 4-(2-hydroxyethyl)-1-piperazineethanesulfonic acid (HEPES), pH 7.8, 25 mM potassium glutamate, 0.25 mM EDTA, and 0.05% Tween-20], 5 mM DTT (dithiothreitol), containing NaCl at concentrations as indicated, 40 U RNase inhibitor (New England Biolabs, M0314S), 0.1 U yeast inorganic pyrophosphatase (New England Biolabs, M2403S), 7 mM ATP, 7 mM CTP, 7 mM GTP, 7 mM UTP, 4 mM CleanCap^®^ reagent AG (Cap-1) (Trilink, N-7113-1) (enzymatic capping for 5.6 kb Cas9-GFP mRNA), and 0.3 M NaCl to achieve a final volume of 20 µl. Where indicated, UTP was replaced with N1-methyl-pseudo UTP (m1ψ). This mixture was incubated at 37°C for 1.5 h.

### Co-tethered transcription

The enzyme–DNA-coupled beads were then exchanged into a transcription mix that includes 1× transcription buffer, additionally containing 5 mM DTT, NaCl at concentrations as indicated, 40 U RNase inhibitor (New England Biolabs, M0314S), 0.1 U yeast inorganic pyrophosphatase (New England Biolabs, M2403S), 7 mM ATP, 7 mM CTP, 7 mM GTP, 7 mM UTP, 4 mM CleanCap® reagent AG (Cap-1) (Trilink, N-7113-1) (except for 5.6 kb Cas9-GFP mRNA, which was enzymatically capped), in a final volume of 20 µl (for mouse studies, the reaction was scaled directly to 250 µl). Where indicated, UTP was replaced with N1-methyl-pseudo UTP (m1ψ). This mixture was incubated at 37°C for 1.5 h using a tube revolver rotator set at 40 rpm (Thermo Scientific™). The reaction was stopped by magnetically pulling down the enzyme–DNA tethered beads, leaving product RNA in solution.

### Enzymatic capping (5.6 kb mRNA)

The Cas9–EGFP mRNA was post-transcriptionally capped with a 7-methylguanylate cap structure (Cap-0) and 2’-O-methylated (Cap-1) at its 5′ end using the Vaccinia Capping System from New England Biolabs (M2080). Briefly, the cleaned-up RNA (10 μg) is denatured at 65°C for 5 min and added to 1× capping buffer (50 mM Tris–HCl, pH 8.0, 5 mM KCl, 1 mM MgCl_2_, 1 mM DTT), 10 mM GTP, 4 mM SAM, 10 U vaccinia capping enzyme, and 50 U mRNA cap 2’-O-Methyltransferase (New England Biolabs, M0366S) and incubated at 37°C for 30 min in 20 μl volume. RNA is ready for use in *in vitro* cell-based assays and *in vivo* mice studies without purification.

### Phosphatase treatment to remove triphosphates

RNAs synthesized using Cap analogs and RNAs enzymatically capped contain residual RNA with a 5′ triphosphate. Elimination of this immune-stimulating triphosphate followed the protocol of Antarctic phosphatase (AnP) (New England Biolabs, M0289S) to remove the phosphates from 5′ ppp-RNA (0.8, 5.6, and 8.6 kb), which are generated during the *in vitro* transcription reaction. One picomole of crude RNA was added to 20 μl of 1× AnP reaction buffer with 5 U AnP and then incubated at 37°C for 30 min.

### Post-transcriptional mRNA polyadenylation

Where indicated, poly(A) tails were added to mRNA (Cas9–EGFP) post-transcriptionally, following the protocol of *E. coli* poly(A) polymerase (New England Biolabs, M0276S). RNA (10 μg) was added to 1× poly(A) polymerase reaction buffer (50 mM Tris–HCl, pH 8.1, 250 mM NaCl, 10 mM MgCl_2_), 1 mM ATP, and 5 U poly(A) polymerase and incubated at 37°C for 30 min. RNA is cleaned up for cell-based assays.

### mRNA desalting for biochemical and cell-based assays

To clean up and concentrate mRNA (0.8, 5.6, and 8.6 kb) for biochemical and cell-based assays, we followed the protocol of the Monarch^®^ Spin RNA Cleanup Kit (New England Biolabs, T2040S).

### mRNA storage

All the mRNA desalted with the NEB cleanup kit was dissolved in the RNA storage buffer, pH 6.7 (Invitrogen, AM7001).

### Reuse and storage of co-tethered enzyme–DNA immobilized beads

The isolated magnetic beads were rinsed once with 200 μl streptavidin wash buffer (20 mM Tris-Cl, pH 7.5, 1 mM EDTA, 0.5 M NaCl, 0.1% Tween-20) and stored in 20 μl of TE buffer (pH 8.0) at 4°C. To reuse the co-tethered enzyme–DNA bead complex, the beads were resuspended in a 1× transcription reaction buffer containing a final 10 mM DTT and incubated at room temperature for 30 min during each repeat-batch transcription.

### RNA seq to validate RNA manufactured from a high salt co-tethered system

Reverse transcription of RNA was conducted using Induro Reverse Transcriptase (New England Biolabs, M0681S) with gene-specific primers, adhering to the manufacturer’s protocol. The reaction was incubated at 55°C for 15 min, followed by a 1 min deactivation step at 95°C. The resulting cDNA (5 µl) was then PCR-amplified in a 1× LongAmp Master Mix (New England Biolabs, M0287S) in a final 50 μl reaction. The resulting PCR product was purified by agarose gel electrophoresis. The purified DNA sample was subsequently sequenced using the Plasmidsaurus Premium PCR service, which employs a non-fragmentation approach for DNA sequencing.

### mRNA transfection and translation assay

HEK293T and RAW264.7 cells were maintained in Dulbecco’s modified Eagle medium, high glucose (Gibco™-11965118), supplemented with 10% fetal bovine serum and 1% penicillin–streptomycin (Gibco™). The cells were seeded 24 h before the experiment in (i) 96-well plates (Nunc™ Edge™; ThermoFisher Scientific) at a concentration of 10^5^ cells/ml in a total volume of 100 µl/well for 0.8 kb NLuc mRNA and (ii) in 6-well plates (USA Scientific) at a concentration of 5 × 10^4^ cells/ml in a total volume of 1 ml/well for 5.6 kb Cas9–EGFP mRNA. The cells were transfected with mRNAs using Lipofectamine™ MessengerMax™ (Invitrogen) as per the manufacturer’s protocol. Briefly, mRNAs were diluted with an appropriate amount of Lipofectamine™ MessengerMax™ (100 ng NLuc mRNA with 0.3 µl Lipofectamine and 5 µg Cas9–EGFP mRNA with 3.5 µl Lipofectamine) and incubated at 25°C for 5 min. The mRNA–lipid complex was then added directly to the media of growing cells. Expression of NLuc mRNA was evaluated 24 h post-transfection using the Nano-Glo® Luciferase Assay System (Promega) as per manufacturer’s recommendation. Briefly, 100 µl of Nano-Glo® Luciferase Assay Substrate (diluted 1:50 with Nano-Glo® Luciferase Assay Buffer) was added to each well containing 100 μl of media. The luminescence was recorded after 5 min using the SpectraMax M5 (Molecular Devices). Background luminescence was measured with RAW264.7 cells, treated as above but without any mRNA transfection.

Expression of Cas9–EGFP was evaluated using flow cytometry 24 h post-transfection. Briefly, cells were washed, collected, and redissolved in 100 µl of 1× PBS. The cells were gated for live population and subsequently for single cell population. EGFP fluorescence was evaluated using the ACEA Novocyte Flow Cytometer by gating the background fluorescence from cells treated with just lipofectamine as a negative control.

### Innate immune response analysis using RT-qPCR

Cellular mRNA from the above was harvested 24 h post-transfection using the Luna^®^ Cell Ready Lysis Module (New England Biolabs). From 2 μl of lysate, the Luna Universal One-Step RT-qPCR Kit was used to generate cDNA of the genes of interest and quantify those cDNAs in a single step, as per the manufacturer’s protocol. The sequences of the primers used for reverse transcriptase-mediated quantitative polymerase chain reaction (RT-qPCR) are in Supplementary Table SI. Cells treated with low-molecular-weight polyinosinic-polycytidylic acid [poly(I:C)] (InvivoGen) were used as a positive control (extracellular dsRNA mimic), and lipofectamine without any mRNA was used as a negative control for RT-qPCR analysis.

The relative fold change was calculated using the (2^−ΔΔCt^) delta–delta Ct method [16]. For RAW264.7 cells, the basal level of interferon beta 1 (IFN-β1) was undetectable using RT-qPCR even after 40 cycles of amplification, and hence the IFN-β1 activity in RAW264.7 cells is measured with respect to poly(I:C)-treated cells and normalized thereafter. Glyceraldehyde-3-phosphate dehydrogenase (GAPDH) was used as the housekeeping gene.

### Quantification of IFN-β1 overexpression in RAW264.7 cells transfected with NLuc mRNA using ELISA

Mouse IFN-beta DuoSet ELISA (Bio-Techne) was used to measure secreted IFN-β1 in media by RAW264.7 cells that were transfected with NLuc mRNA (500 ng/well in a 96-well plate, 10 000 cells/100 µl well). Briefly, a 96-well plate was coated with diluted capture antibody (rat anti-mouse IFN-β capture antibody) at room temperature overnight. The wells were then thoroughly washed with wash buffer (0.05% Tween-20 in PBS, pH 7.2–7.4) three times before blocking the wells with 1% BSA (in PBS, pH 7.2–7.4). Samples or standards were added to it after washing the plates with wash buffer and incubated at room temperature for 2 h. This was followed by washing and incubating with detection antibody (Biotinylated Rat Anti-Mouse IFN-β) at room temperature for 2 h. Streptavidin-HRP was then added after washing and incubated at room temperature for 20 min. Substrate solution (1:1 mixture of H_2_O_2_ and tetramethylbenzidine) was then added and incubated for 20 more min at room temperature. Finally, 0.66 N H_2_SO_4_ was added to stop the reaction, and OD was recorded at 450 nm and 570 nm. A standard curve was generated using the provided standard to accurately measure the IFN-β1.

### Synthesis of NLuc mRNA lipid nanoparticles

To deliver the NLuc mRNA *in vivo* in mice, the mRNA was encapsulated in ionizable lipid nanoparticles (LNPs) using a microfluidic mixing technique. In brief, m1ψ-modified NLuc mRNA was dissolved in 12.5 mM sodium acetate, pH 4.5, at a concentration of 0.075 mg/ml, making up the aqueous mRNA phase for the nanoassembly. For the lipid phase, the total amount of lipids was calculated by keeping a 1:10 mass ratio of NLuc mRNA to ionizable lipid, using an overall lipid formulation mole ratio of 50% ionizable lipid, 10% phospholipid, 38.5% cholesterol, and 1.5% PEGylated lipid, respectively. For the specific lipids used in the study, we used the ionizable lipid SM-102, phospholipid 1,2-dioleoyl-sn-glycero-3-phosphoethanolamine, cholesterol, and the PEGylated lipid 1,2-dimyristoyl-rac-glycero-3-methoxypolyethylene glycol-2000 (DMG-PEG 2000). Each lipid was dissolved in 200-proof ethanol so that the entire lipid mixture made up one-third the volume of the mRNA solution. After preparing both mRNA and lipid mixtures, they were loaded in disposable syringes and passed through a Y-channel CHP-MIX-3 NanoParticle synthesis micro-mixer chip (PreciGenome) at a total flow rate of 2.5 ml/min, while keeping the mRNA to lipid flow rate ratio at 3:1. After microfluidic mixing, the resultant turbid nanoparticle solution was left to sit at room temperature for 15 min to complete the assembly and then concentrated and buffer exchanged into 1× PBS. For this, the nanoparticles were diluted in 1× PBS and spun down using Amicon 15 ml 10000 MWCO centrifugal filters at 3000 × *g* for 45 min, followed by a second 1× PBS dilution and spin, to remove as much ethanol and acetate as possible. The resultant concentrated LNP was then used for subsequent characterization and encapsulation efficiency studies for use in the *in vivo* mouse experiment. For the *in vivo* experiment, a nascent (empty) nanoparticle was used in each of the analyses. To make this, we used the same microfluidic mixing protocol as described previously, using the same lipid type ratios of 50% ionizable lipid, 10% phospholipid, 38.5% cholesterol, and 1.5% PEGylated lipid, except in this case, no mRNA was added to the aqueous sodium acetate phase before microfluidic assembly. Otherwise, the same protocol was followed.

### LNP characterization—size and zeta potential

For nanoparticle characterization, the resultant LNPs were diluted 200-fold in either 1× PBS or water for size and zeta potential measurements, respectively, using a Malvern Nanozetasizer ZS90. After loading the samples in DLS or zeta cuvettes, measurements were taken. Unless otherwise stated, LNP size was determined by the Nanozetasizer intensity Z-average, and the particle size distribution was also determined using the Nanozetasizer intensity stats table.

### LNP characterization—encapsulation efficiency

To determine the encapsulation efficiency of NLuc in the LNPs for proper dose administration in the *in vivo* experiments, the Invitrogen Quant-iT RiboGreen assay was used. In brief, 3 μl of mRNA-LNPs were dissolved in 300 μl of 1× Tris–EDTA (TE) buffer, either with or without the addition of 2% (v/v) Triton-X-100 surfactant, which was used to break apart the lipid bilayer and release the encapsulated mRNA cargo. After diluting the LNPs in the resultant solutions, they were vortexed and then aliquoted into 96-well black/clear-bottom plates, followed by the addition of 100 μl RiboGreen reagent, prepared according to the manufacturer’s protocol. After 3–5 min of incubation, the plate was analyzed in a plate reader to determine the 480/520 fluorescence intensity of the samples. Each sample fluorescence was subtracted from the blank RiboGreen in buffer signal. To calculate encapsulation efficiency, a ratio of the 1× TE sample without Triton fluorescence, denoting unencapsulated RNA, to 1× TE with Triton fluorescence, denoting the total unencapsulated as well as encapsulated RNA, was taken. Subtracting this value from 1, we were able to determine the amount of encapsulated mRNA in the LNPs.

### *In vivo* assessment of NLuc mRNA transfection efficiency and immunogenicity in a subcutaneous Matrigel plug mouse model

To test the immunogenicity and transfection/expression efficiency of the NLuc mRNAs *in vivo*, we developed a Matrigel plug immune cell infiltration and bioluminescence mouse model. We selected a subcutaneous Matrigel plug model to simultaneously assess mRNA translation and immune cell recruitment, as it allowed sufficient cell recovery for reliable flow cytometry. While intramuscular delivery is more typical for vaccines, our goal here was a proof-of-concept comparison of RNA synthesized through a traditional protocol versus RNA synthesized with the co-tethered system. To begin, male 6–8-week-old C57BL/6 mice (Charles River Laboratories) were shaved on their back right flank and then subcutaneously injected with a 200 μl mixture of Corning Matrigel ECM matrix combined with 30 μg doses of mRNA-LNPs encapsulating either co-tethered transcribed or traditional batch transcribed m1ψ-modified NLuc mRNA or the representative nascent (empty) LNPs. The selection of a 30-µg subcutaneous dose for this *in vivo* study was informed by several key considerations including dosing reported in existing literature [17, 18], our prior experience in mRNA delivery [19], and the strategic goal of balancing immune response sensitivity against potential oversaturation by the LNP delivery system. The Matrigel mixture consisted of roughly 80%–85% by volume Corning Matrigel and ∼15%–20% by volume LNP solutions, depending on the final volume of the LNP treatments after concentration. The mixture was kept on ice before and during administration to prevent premature coagulation and polymerization of the Matrigel. After injection, the Matrigel was allowed to solidify, and the mice were monitored and imaged using a Perkin Elmer *In Vivo* Imaging System (IVIS) to assess bioluminescent activity indicative of NLuc expression. For this protocol, the mice were anesthetized under isoflurane at 6, 16, 24, 48, and 72 h, kept in the IVIS under a stable supply of isoflurane and oxygen through the nose cone apparatus, and then subcutaneously injected with 10 μM of NanoGlo^®^ Fluorofurimazine *In Vivo* Substrate. The NLuc substrate was injected near, but not directly into, the Matrigel plug to prevent any off-target or unexpected immunogenicity induced by the substrate. The flanks of the mice were then lightly and carefully massaged for 3–5 min. Finally, the mice were imaged using the luminescence settings on the IVIS system, and the bioluminescent ROI counts for the flanks of each mouse were quantified using the Perkin Elmer Living Image software at each time point, using the same bioluminescence scale for each image and time point. After the final 72 h bioluminescence time point, the mice were euthanized, and the Matrigel plug was excised for further flow cytometric analysis.

### *In vivo* immunoprofiling using flow cytometry

After the Matrigel plug excision, the infiltrated immune cells were extracted for immunoprofiling by flow cytometry. For this protocol, the excised Matrigel plugs were diced into small pieces and homogenized by incubation with 1 mg/ml of Collagenase Type IV and 0.1 mg/ml of DNase 1 in basal RPMI media at 37°C for 1 h, followed by dilution to a total volume of 3 ml in RPMI and passage through 70 μm cell strainers. Next, the resultant cell suspension was centrifuged at 1350 rpm for 5 min. The supernatant was then aspirated, and the cell pellet was washed twice with cold RPMI before resuspending it in 3% FACS buffer (1× PBS with 3% fetal bovine serum). Next, the cells were counted using a hemacytometer to ensure proper cell density for antibody staining (10^6^ cells in 100 μl), and portions of each sample were aliquoted into two separate flow panels in microcentrifuge tubes, panel A and panel B. The cell sample in panel A was used to assess monocyte-derived infiltrated macrophages, dendritic cells, and their co-stimulatory receptor expression indicative of immune activation state, staining with BV605 CD45.2 (immune cells), APC F4/80 (macrophages), BV421 CD11c (dendritic cells), FITC CD80, and PE/Dazzle594 CD86 (co-stimulatory receptors), according to BioLegend standard protocols. Panel B was used to assess neutrophil and T-cell infiltration, staining with BV605 CD45.2 (immune cells), PE Ly6-g (neutrophils), and BV421 CD3 (T cells), also according to BioLegend protocols. To ensure proper lower limit of expression gating for each surface marker, Fluorescence Minus One and single-stain controls were conducted by combining a portion of each sample into a pooled sample that was aliquoted and stained according to each FMO/single-stain control. After 30 min of staining at 4°C, the cells were pelleted, the supernatant was aspirated, and then the cells were washed with FACS buffer before finally resuspending the pellet in 100 μl FACS buffer to run the sample using an ACEA Novocyte 3000 Flow Cytometer. Gating and analysis were conducted using NovoExpress software.

## Results

In recent studies describing a functionally co-immobilized RNA polymerase and promoter-containing DNA co-catalyst, DNA with chemical “handles” was prepared via the introduction of upstream and downstream functional groups contained within PCR primers [7]. In extending this work to longer RNAs and introducing an alternative to PCR, we demonstrate here the incorporation of functional handles via a fill-in approach applicable to DNA from any source.

We used plasmid DNA constructs having 806, 5659, and 8647 bp sequences encoding 789, 5642, and 8630 base length mRNA to demonstrate the plasmid-compatible enzyme–DNA co-tethered transcription.

### Plasmid-compatible co-tethering approach

**Figure 2.**
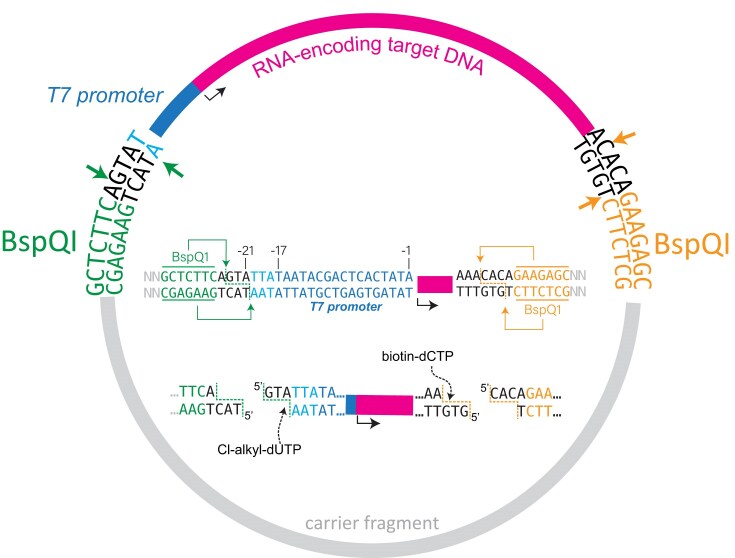
Design for asymmetric labeling of mRNA encoding DNA. Two unique BspQI recognition sites are strategically positioned flanking the RNA-encoding region of the plasmid. BspQI cleavage generates defined 3′ recessed overhangs, enabling asymmetric fill-in with modified nucleotide analogs, such as Cl-alkyl-dUTP and biotin-16-dCTP.

As an alternative to PCR, in the current work, we cleave DNA using “scarless” BspQI restriction sites, as shown in Fig. 2. The resulting 5′ overhanging ends are designed such that a one-pot Klenow fill-in reaction incorporates the chemical handles asymmetrically into (only) the DNA fragment containing the RNA-encoding sequence. The Cl-alkyl-dUTP modifies DNA at the 3′ end upstream of the promoter and is then ready for coupling to HaloTag^®^ T7 RNA polymerase to generate a functionally co-tethered enzyme–DNA complex. The T7 promoter extends ~17 base pairs upstream (−17) of the site where transcription starts (+1). In the sequence shown in Fig. 2, Cl-alkyl-dUTP will incorporate outside of the promoter, at (only) position −21 (with the exception of 5.6 kb Cas9–EGFP mRNA, where the incorporation happens at position −18).

In the current system, a functionally co-tethered enzyme–DNA complex is attached to a streptavidin-coated surface via incorporation of biotin at the downstream end of DNA, using biotin-16-dCTP, as shown in Fig. 2. A strength of this labeling approach is that intermediate purifications of RNA-encoding DNA templates from carrier fragments are not required. BspQI-cleaved DNA was labeled in a one-pot reaction using Cl-alkyl-dUTP and biotin-16-dCTP, incorporating these modified nucleotides opposite complementary adenine and guanine bases, respectively. The design labels only the DNA fragment containing the RNA-encoding sequence. The resulting mixture can be loaded directly onto streptavidin-coated magnetic beads, and the plasmid carrier fragment can be readily washed away. Characterization of the fill-in reaction using a short DNA fragment (Supplementary Fig. S1) demonstrates efficient label incorporation.

### Synthesis of short RNA transcripts demonstrates approach

As an initial demonstration of the approach, we started with a synthetic 87 bp DNA, encoding a 70 base length RNA, and incorporating Cl-alkyl-dUTP and biotin-16-dCTP as above (Fig. 2). This system allows direct observation of RNA self-primed synthesis, as shown in the control in Supplementary Fig. S4A. The free enzyme and DNA system shows the formation of higher-molecular-weight RNA species. Since the extension is distributive, multiple RNA re-bindings in the free system at 0 M added NaCl lead to very long RNAs (the smear in Supplementary Fig. S4A). As in earlier studies, the addition of salt decreases these impurities but decreases overall yield as well.

As expected, the co-tethered transcription system, even with no added NaCl, significantly reduces long extensions and, with increased salt, eliminates even short additions (Supplementary Fig. S4B). In contrast to the free system, good yield is maintained up to at least 0.5 M added NaCl.

We also tested the reusability of the co-tethered system by repeat batch synthesis (at 0.3 M added NaCl) over 10 days (Supplementary Fig. S4C). For overnight storage at 4°C, the immobilized beads were incubated in TE buffer (pH 8.0), and 5 mM final DTT was added to the storage buffer to ensure optimal activity. The ten-repeat batch syntheses demonstrated excellent yield. We then synthesized longer RNAs (0.8, 5.6, and 8.6 kb) from plasmid DNA. To demonstrate the efficient synthesis of highly pure mRNA products, we used the design shown in Fig. 2 to prepare plasmid DNAs encoding 0.8, 5.6, and 8.6 kb mRNAs. The 0.8 kb mRNA encodes NLuc, while the 5.6 kb mRNA encodes a human codon-optimized SpCas9 with a C-terminal bi-partite NLS, 3× Flag tag, and P2A–EGFP fusion. The 8.6 kb mRNA encodes an ELYS–Emerin–EGFP (in that order) fusion. As previously described, chemical handles were appended using a one-pot fill-in reaction, and the RNA-encoding DNA fragments were then immobilized on streptavidin-coated magnetic beads. All constructs, except the 5.6 kb Cas9–EGFP mRNA, include an encoded 60–120 base poly(A) tail (see Supplementary data). The final concentrations in the solution-phase reaction were 20 nM RNA polymerase and 100 nM DNA.

**Figure 3.**
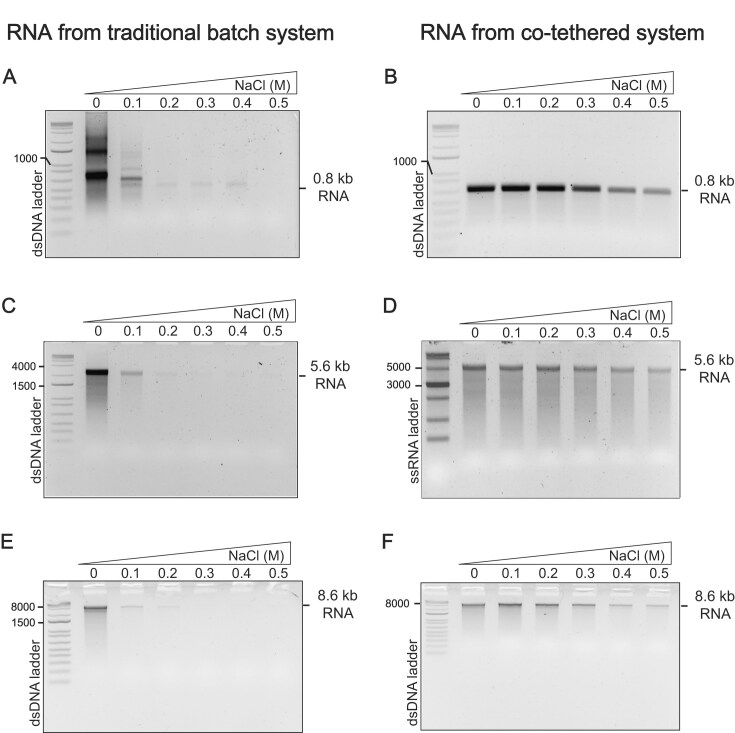
Run-off transcription analysis on a 2% agarose gel of RNA. (**A**), (**C**), and (**E**) show transcription by free DNA polymerase from 0.8 kb (NLuc), 5.6 kb (Cas9–EGFP), and 8.6 kb (ELYS–Emerin–EGFP) templates, respectively, resulting in substantial amounts and lengths of dsRNA impurities under low-salt conditions and reduced RNA yields at higher salt. In contrast, (**B**), (**D**), and (**F**) depict transcription by functionally co-tethered complexes from the same templates, producing fewer dsRNA impurities and maintaining good RNA yields even at higher salt concentrations.

The results shown in Fig. 3 demonstrate the expected behavior for RNAs of lengths 0.8, 5.6, and 8.6 kb. For all three, solution-phase transcription [panels (A), (C), and (E)] is highly sensitive to salt, while co-tethered transcription [panels (B), (D), and (F)] is salt resistant up to ~0.3 M added NaCl. The salt sensitivity of solution-phase transcription is somewhat higher for these longer lengths relative to 70-base RNA synthesis, which likely reflects the fact that transcription of longer-length RNAs necessarily uses lower concentrations of the encoding DNA construct (and enzyme), which effectively weakens binding. For the solution reactions, 1.0, 6.5, and 10 µg of linearized plasmid DNA were used to transcribe the 0.8, 5.6, and 8.6 kb mRNA constructs, which correspond to 0.022, 0.061, and 0.060 µM promoter, respectively. RNA polymerase was present at 0.02 µM. These contrast with the 70-base RNA synthesis (Supplementary Fig. S4), which employed 0.87 µM promoter and 0.2 µM RNA polymerase.

### Repeat batch synthesis of 0.8 to 8.6 kb mRNAs

**Figure 4.**
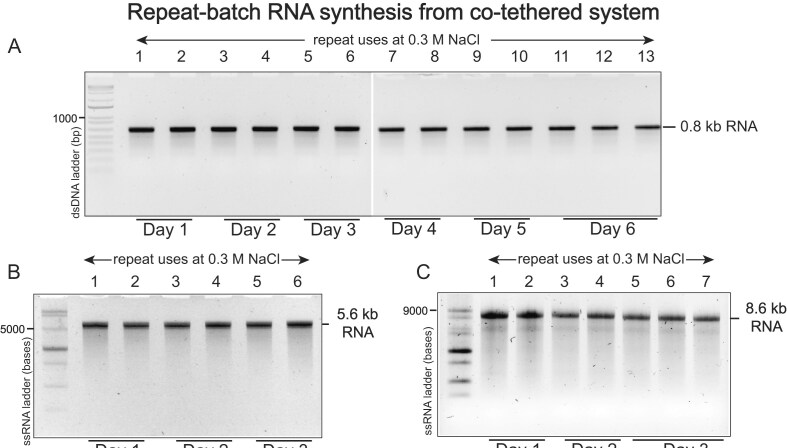
Repeat-batch run-off transcription at 0.3 M salt using the same co-tethered enzyme–DNA complex for (**A**) 0.8, (**B**) 5.6, and (**C**) 8.6 kb RNA transcripts, analyzed on a 2% agarose gel. The results demonstrate consistent transcription performance across template sizes under high-salt conditions.

RNA manufacturing in single batches suffers from low yields and high costs due to the disposal of DNA templates and RNA polymerase. Here, our co-tethered enzyme–DNA complex is immobilized on streptavidin beads, making it ideal for repeat-batch and flow manufacturing. Our data show mRNA synthesis of 0.8 kb (Fig. 4A), 5.6 kb (Fig. 4B), and 8.6 kb mRNA (Fig. 4C) in >6 batches, reusing the same enzyme–DNA co-tethered complex at 0.3 M NaCl. It can be calculated that current RNA manufacturing from free enzyme–DNA partners allows one set of *in vitro* transcription reactions. The co-tethered enzyme–DNA immobilized complex enables multiple repeat batches from the same enzyme–DNA partners, demonstrating the economic viability of RNA manufacturing. The 10 repeat-batch syntheses of 0.8 kb RNA from the same co-tethered enzyme–DNA system yielded more RNA (500 μg) compared to 120 μg from the free enzyme–DNA system.

Our analysis confirmed that co-tethering T7 RNA polymerase to plasmid DNA did not interfere with the synthesis of the correct RNA product. RNA produced from both Cas9–EGFP (5659 bp) and ELYS–Emerin–EGFP (8647 bp) plasmids using the co-tethered system was successfully reverse transcribed, and the cDNA was PCR amplified. Gel electrophoresis revealed the expected band, which was subsequently purified and sequenced. The sequences demonstrated that the consensus sequence matched the expected encoded RNA from the plasmid, validating the robustness of our co-tethered system in synthesizing long RNA.

### Translatability of 0.8 kb NLuc mRNA

The above results demonstrate that, as hypothesized by the highly effective local concentration of binding partners in the tethered system, the functionally co-tethered polymerase–DNA system displays significant salt tolerance. As the target RNA’s length increases, RNA contamination by RNA containing double-stranded regions (dsRNA) is increasingly difficult to quantify (high background from RNA with its own short, and therefore non-immunogenic dsRNA regions). While dot blots and ELISA assays using antibodies to detect dsRNA are widely used, they are well-known to be of relatively low sensitivity. To demonstrate low immunogenicity in a more relevant assay, below we measure both reporter expression (the immune response downregulates translation) and innate immune response stimulation in two different cell lines.

The structural design of mRNA vaccines closely resembles that of eukaryotic mRNA, comprising a single-stranded RNA molecule with a 5′ cap structure, a 3′ polyadenylated poly(A) tail, and a central open reading frame flanked by 5′ and 3′ untranslated regions. In the following, we characterize mRNA constructs of different lengths, containing either native U or m1ψ. In each case, we compare mRNA synthesized by a commonly used high-yield commercially available kit (traditional batch synthesis) to that synthesized by the enzyme–DNA co-tethered approach described here. The mRNAs were then minimally processed, as indicated, and then directly transfected into (macrophage) RAW264.7 cells.

**Figure 5.**
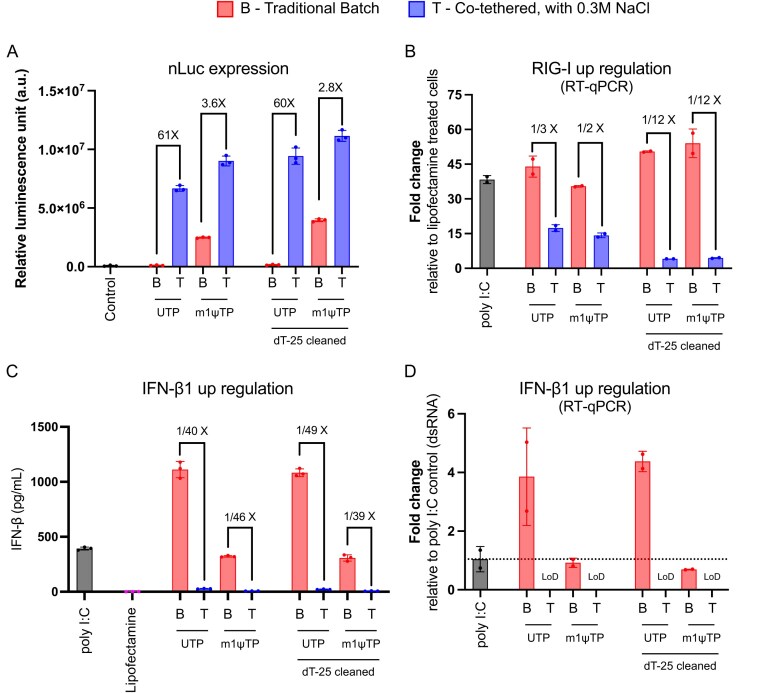
Expression and innate immune response in macrophage cells. (**A**) Nanoluciferase activity of 0.8 kb NLuc mRNA transfected into RAW264.7 cells. mRNA was transcribed under traditional batch (B) or high salt, co-tethered (T) conditions. One hundred nanograms of mRNA was transfected into RAW264.7 cells. mRNA was synthesized with substrate UTP or m1ψ, as indicated. Luminescence was assayed 24 h post-transfection. The same mRNAs were then purified using oligo d(T)_25_ beads (pull-down) and re-assayed. (B–D) Expression of genes known to be upregulated by dsRNA. (**B**) RIG-I is a cytosolic receptor that can bind to dsRNA and gets upregulated. (C, D) IFN-β1 is a type-I interferon that measures overall immune response, and overexpression of the IFN-β1 gene was analyzed by both ELISA (**C**) and RT-qPCR (**D**).

In the studies presented in Fig. 5, assaying 0.8 kb mRNA encoding NLuc, 24 h post-transfection, luciferase assay substrate (furimazine) was added to the cells with cell lysing buffer. The expression results show the luciferase activity of each batch of mRNA produced by the co-tethered or batch approach. Consistent with our earlier work, mRNA made by the co-tethered approach at 0.3 M NaCl shows much higher (61× for U-incorporation) expression (Fig. 5A). Substitution of U by m1ψ is known to reduce dsRNA production [20–24], and consistent with that, expression increases dramatically in the batch-synthesized mRNAs containing m1ψ. The co-tethered approach benefits much less from m1ψ incorporation, consistent with its containing substantially lower levels of dsRNA impurities.

Oligo d(T)_25_ affinity clean-up is used to select RNA only with poly-A tails, as truncated fragments will not contain the affinity target. In principle, this purification will also remove RNA containing extensive dsRNA segments (large enough to preclude oligo d(T)_25_ binding in a 60-base poly(A) tail). The results in Fig. 5A show that such a clean-up increases protein production across the board, but to a relatively small extent.

Immune response results show that high levels of protein expression are achievable in macrophages without using modified uridines. Incorporation of m1ψ provides a layer of protection against Toll-like receptor (TLR)-mediated immune system activation to the mRNA; however, its usage presents challenges such as +1 ribosomal frameshifting [25] and an altered translation process due to increased ribosome pausing [26, 27]. Our data suggest that the IVT process with canonical NTPs can be strategically optimized to produce no to low dsRNA content at the time of synthesis, which can reduce the dependency on using m1ψ without losing much of the benefits that come with the usage of m1ψ. This is further investigated in the next section.

### Low immunogenicity of 0.8 kb NLuc mRNA

Since the innate immune response downregulates protein expression in the cell [28, 29], the above results indirectly demonstrate reduced immune response in mRNA synthesized at high salt. To more directly evaluate the interpretation, we assayed for upregulation of markers known to respond to immune stimulation. RT-qPCR shows upregulation of the innate immune response marker IFN-β1, a type-I interferon that is increased in response to dsRNA impurities [30], and upregulation of RIG-I, a cytosolic receptor that is involved in sensing multiple aspects of viral RNA [31–33].

The results shown in Fig. 5D demonstrate that batch-synthesized mRNA elicits a measurable upregulation of IFN-β1 expression, consistent with previous findings [7]. In contrast, mRNA synthesized at 0.3 M NaCl using the co-tethered system exhibits IFN-β1 levels below the limit of detection. As previously observed, incorporation of m1ψ attenuates but does not fully abrogate the immune response in batch-synthesized mRNA [34, 35]. Oligo d(T)_25_ affinity purification yields only a modest reduction in IFN-β1 expression. These findings are corroborated by IFN-β1 protein quantification via ELISA, which reveals a consistent trend (Fig. 5C).

As shown in Fig. 5B, parallel assays reveal a marked upregulation of the cytosolic receptor RIG-I. This response is substantially attenuated in mRNA synthesized at 0.3 M NaCl using the co-tethered system, compared to traditional batch-synthesized mRNA. Consistently, substitution of uridine with m1ψ results in only a modest reduction in RIG-I activation.

Overall, mRNA containing m1ψ does not significantly alter RIG-I–mediated immune activation; however, it reduces type I interferon responses by evading pattern recognition receptor-mediated detection, particularly through TLRs.

### Expression of 5.6 kb Cas9–EGFP mRNA in different cell types

For the 5.6 kb Cas9–EGFP (and 8.6 kb ELYS–Emerin–EGFP) mRNA produced using the co-tethered system, RNA was reverse transcribed, PCR amplified, and sequenced. The results in each case confirm the sequence identity.

**Figure 6.**
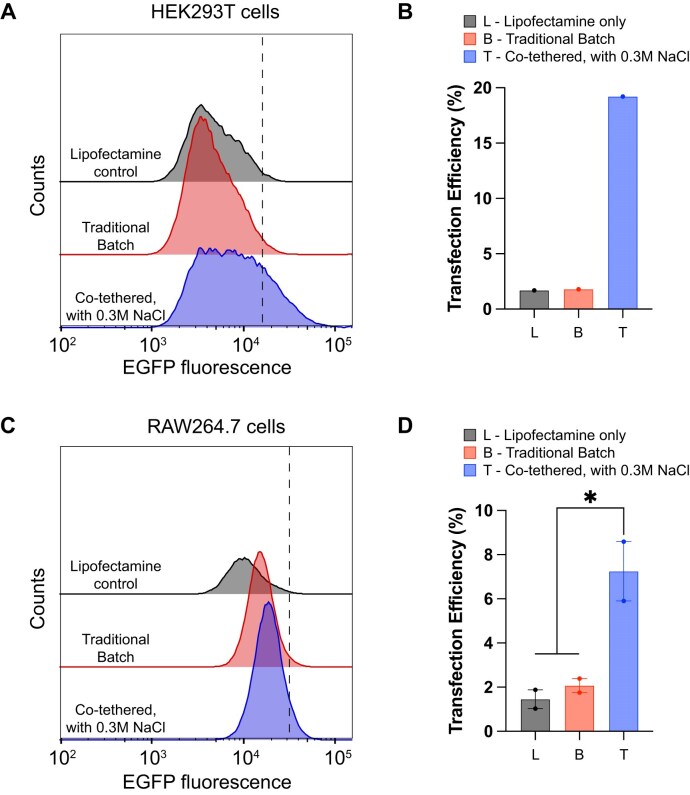
Flow cytometer (histogram) analysis of 5.6 kb Cas9–EGFP (containing m1ψ) mRNA translation. (**A**) Quantitative FACS analysis of mRNA transfected into HEK293T cells. (**B**) Quantification of those data. (**C**) FACS analysis of RAW264.7 (macrophage) cells. (**D**) Quantification of the RAW264.7 data. Transfection efficiency with biological replicates (*n* = 2) is calculated from their corresponding histograms. Tukey’s multiple comparison test is performed, and statistical significance with P < .05 (*) is illustrated.

To test the robustness of the above results, we next tested the translatability of a 5.6 kb transcript that encodes GFP from a Cas9–EGFP fusion construct. As above, Cas9–EGFP mRNA incorporating m1ψ was synthesized using either the traditional batch approach or via co-tethered synthesis at 0.3 M added NaCl. As shown in Fig. 6, expression was measured using flow cytometry in HEK293T cells and RAW264.7 cells. After gating for live single-cell population, we have seen the number of cells producing EGFP was ∼2–25 times more for cells treated with Cas9–EGFP transcript made by tethered approach at 0.3 M added NaCl compared to the cells made using traditional batch synthesis. The number of positive cells counted for the EGFP signal was gated based on the background cell fluorescence [panels (A) and (C)] to eliminate false-positive responses.

The observed inability of traditional batch-synthesized Cas9–EGFP mRNA, lacking substantial purification, even with m1ψ, to translate the protein in cells [36] is consistent with our previous results with 0.8 kb NLuc mRNA (Fig. 6A), as well as reports from our group [7] and others [34]. Extensive purification steps to remove dsRNA contaminants are the primary path to get any IVT mRNA made using traditional batch synthesis for effective translation by the cells.

### Innate immune response of 5.6 kb Cas9–EGFP mRNA in macrophages

**Figure 7.**
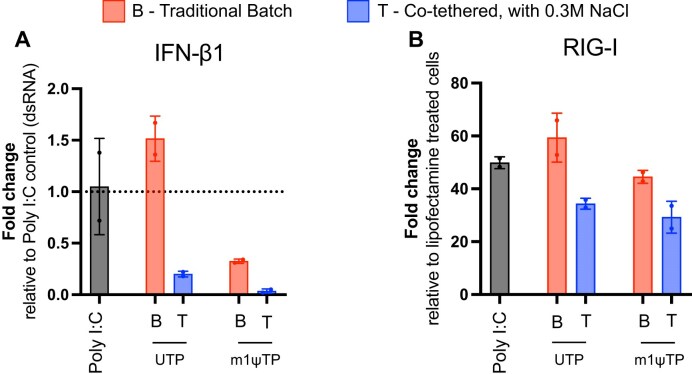
RT-qPCR assay to evaluate innate immune activation in RAW264.7 cells 24 h after transfecting with 5.6 kb Cas9–EGFP mRNA (**A**) interferon-β1 response with respect to poly(I:C) depicts overall innate immune activation from the Cas9–EGFP mRNA transfection, and (**B**) overexpression of RIG-I captures cytosolic dsRNA experienced by the cells after mRNA transfection.

Transfection with 5.6 kb Cas9–EGFP mRNA elicited a much lower type-I interferon (IFN) response profile with the mRNA synthesized using the co-tethered approach compared to that of the mRNA made using a traditional commercial kit (Fig. 7A). Incorporation of m1ψ into traditional batch-synthesized mRNA led to an ∼3-fold reduction in IFN-β1 overexpression. Notably, mRNA synthesized via the co-tethered method without base modification showed over a three-fold decrease in IFN-β1 expression compared to unmodified traditional batch-synthesized mRNA (Fig. 7A), indicating superior mRNA quality and effective dsRNA-free synthesis of long transcripts. Furthermore, inclusion of m1ψ in mRNAs made using tthe co-tethered approach further attenuated IFN-β1 expression to near basal levels, likely due to evasion of TLR-mediated recognition, thereby minimizing innate immune activation [37].

We also quantified RIG-I response, following transfection of cells with Cas9–EGFP mRNA synthesized using both UTP and m1ψ, as well as mRNA generated via the co-tethered approach (Fig. 7B). Both UTP- and m1ψ-containing mRNAs exhibited significantly lower RIG-I expression compared to their traditional batch-synthesized counterparts and to poly(I:C), a synthetic dsRNA mimic. These findings are consistent with our previous observations using NLuc mRNA and further support the effectiveness of the co-tethered strategy in generating high-quality, dsRNA-free mRNA. This approach enables the synthesis of long transcripts (e.g. 5.6 kb Cas9–EGFP mRNA) directly from plasmid DNA while minimizing innate immune activation.

### Expression of NLuc mRNA in a subcutaneous Matrigel plug mouse model

**Figure 8.**
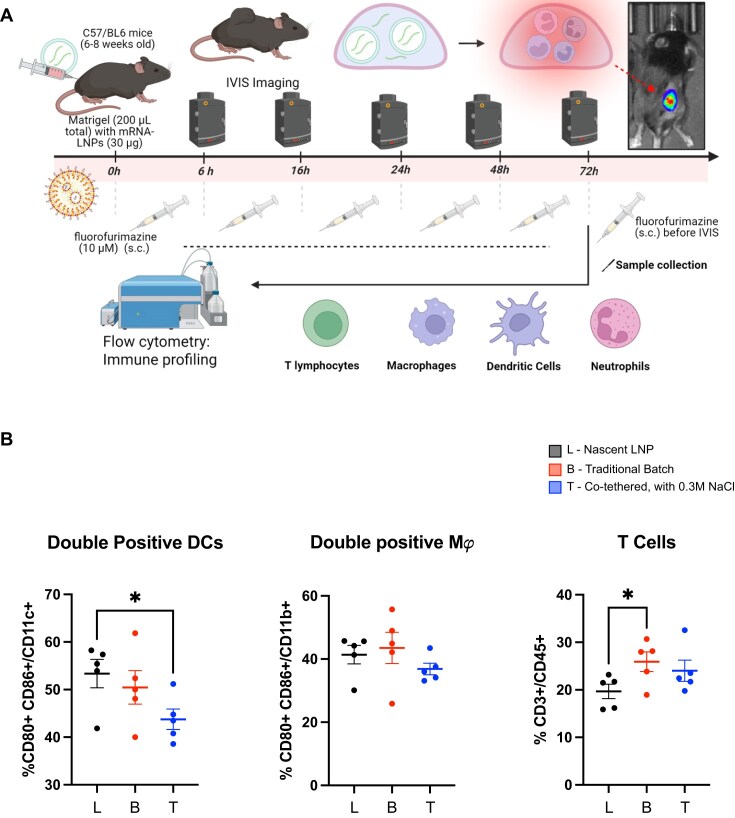
A new Matrigel plug model assay for expression in mice. Assessment of co-tethered-transcribed NLuc mRNA encapsulated in LNPs for delivery in an immune cell infiltration Matrigel plug mouse model. (**A**) Schematic overview of the Matrigel plug immune infiltration and bioluminescence model. In brief, male 6–8-week-old C57BL/6 mice were subcutaneously inoculated in their right flanks with a mixture of Matrigel containing nascent (empty) LNPs or LNPs containing NLuc mRNA produced through either co-tethered or traditional batch transcription. Nanoluciferase activity was assessed 6, 16, 24, 48, and 72 h after administration by subcutaneous injection with fluorofurimazine. At 72 h, the mice were euthanized, and the Matrigel was excised for flow cytometric analysis of immune cell infiltration. (**B**) Percentage of CD11c^+^ cells, F4/80^+^ cells expressing both CD80 and CD86, indicative of active or “matured” dendritic cells and macrophages, respectively, and percentage of T cells out of all immune cells infiltrated in the Matrigel. Unless otherwise stated, data shown are ± SEM (*n* = 5). Statistical analysis was performed using unpaired *T-*test. Detailed gating strategy can be found in Supplementary Fig. S8.

As a final test of the quality of mRNA produced directly from the co-tethered system, we assessed its performance in an *in vivo* setting in mice. Specifically, we aimed to design *in vivo* studies that would enable the simultaneous assessment of immunogenicity and mRNA expression in the tissue. For this, we developed a subcutaneous Matrigel plug model in C57BL/6 mice, allowing monitoring of immune cell recruitment to the Matrigel after co-injection with LNPs carrying m1ψ-modified NLuc mRNA, produced either through a co-tethered or traditional batch process, or nascent (empty) LNPs as a stable control. To produce the treatment groups for this study, we encapsulated the NLuc transcription products in ionizable LNPs (Supplementary Fig. S5A). For the nascent LNP control, the LNPs were made without any mRNA cargo in the acetate buffer during microfluidic assembly. After nano assembly, the LNPs were characterized for size and encapsulation efficiency (Supplementary Fig. S5B and C) by dynamic light scattering and the Quant-it RiboGreen assay, respectively, before administration in the developed subcutaneous Matrigel model, schematically depicted in Fig. 8A.

The *in vivo* studies yielded interesting insights into how mRNA-LNPs and their mRNA cargo affect immune cell recruitment. Overall, we observed a significant degree of immune cell infiltration in the nascent LNP control alone, where ~40% of infiltrated cells in the Matrigel were CD45.2^+^ immune cells (Supplementary Fig. S6A). Among these infiltrated immune cells, we categorized ∼40% of the cells to be Ly6G+ (neutrophils), ∼15% of them to be F4/80+ (macrophages), and ∼10%–15% of them to be CD11c+ (dendritic cells) (Supplementary Fig. S6B–D). These percentages suggest a strong initial neutrophil recruitment, which persisted throughout the course of the 72 h study. We also did not observe any statistical increase or decrease in these cell populations with either mRNA-encapsulating LNP group, whether co-tethered- or traditional batch-synthesized NLuc transcript. These results altogether suggest that, at least at the systemic level, mRNA-LNP immunogenicity and immune cell recruitment are largely dominated by the inflammatory ionizable lipid in the nanoparticles. However, we did observe a few statistically significant findings that suggest that mRNA from the co-tethered transcription system is immunogenically less taxing compared to mRNA from traditional batch synthesis. For example, when we assessed the co-expression of CD80 and CD86 on the surface of dendritic cells, we observed a statistically significant 10% decrease in double-positive DCs, known as immune-active or “mature” DCs, for the LNPs containing co-tether-synthesized NLuc mRNA compared to nascent LNPs, which showed no statistical difference when compared to the LNP group with traditional batch-synthesized NLuc mRNA (Fig. 8B). We also observed a marginal 5% decrease in double-positive macrophages, although this was not found to be significant (Fig. 8B). Although in general, we did not observe a decrease in innate immune response from the LNP group containing co-tethered-synthesized NLuc mRNA across the board, we did observe that the LNPs containing co-tethered-synthesized NLuc mRNA limited adaptive immune responses in terms of T-cell infiltration. Whereas the LNPs containing co-tethered-synthesized NLuc mRNA did not induce any significant increase in CD3^+^ T-cell population, LNPs with traditional batch-synthesized NLuc mRNA did show statistically higher T-cell infiltration compared to nascent LNPs (Fig. 8B). Although small differences, these statistically relevant decreases in inflammatory dendritic cells and T-cell infiltration suggest that the co-tethered system could shift how the innate immune system bridges into an adaptive immune response, potentially affecting the crosstalk between antigen-presenting cells and T cells. It is likely that this shift in adaptive immunity is caused by mRNA cargos affecting the developing Matrigel innate immune environment early in the study, which may not produce robust differences in innate immune cell levels at the final 72 h timepoint but could induce a lasting effect on the shaping of adaptive immune responses, which we observed. This interpretation is supported by several literature sources, which have highlighted that even minor changes in early cytokine milieu and innate immune signalling, whether from differences in mRNA modifications or nanoparticle formulations, can elicit strong shifts in adaptive immunity, CD4^+^ T cell upregulation, and antigen presentation/recognition [38–40].

Overall, while we were able to observe significant reductions in active DCs and T cells from the co-tethered transcription group, the ionizable lipid nanocarriers themselves were the key drivers of immune cell recruitment in the Matrigel model. This finding is supported by several previous studies, which have suggested a substantial effect of ionizable lipids themselves on immunogenicity. For example, it has been widely studied that ionizable LNPs used in the COVID-19 vaccines promote rapid neutrophil infiltration and inflammation [41–44]. A recent study has also demonstrated that ionizable LNPs, including SM-102 LNPs, elicit strong NF-κB and IRF immune responses through TLR4 activation, also leading to an increase in CD86 surface expression in THP-1 monocytes [45]. Our previous work has also discerned that empty ionizable LNPs can activate the NLRP3 inflammasome in a lipid composition-dependent manner [46]. These previous literature sources would together explain the high neutrophil and overall immune cell counts that we observed in the Matrigel from just nascent LNP alone and why at least in the overall systemic immune recruitment, the mRNA transcription method did not yield a significant difference with these cell types.

**Figure 9.**
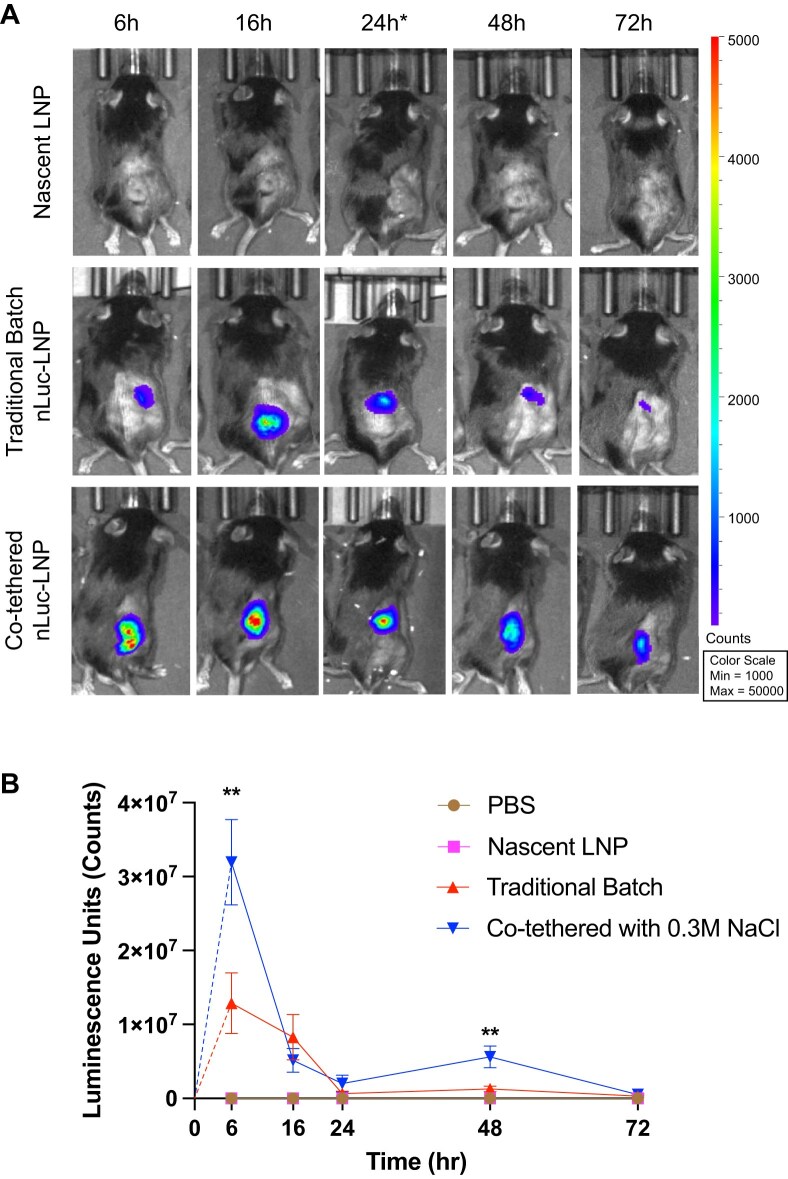
Bioluminescence activity assessment of co-tethered NLuc encapsulating LNPs in the Matrigel model. (**A**) Representative time-point images of the Matrigel flank bioluminescence taken using the IVIS after a 10 μM subcutaneous injection of fluorofurimazine substrate in each mouse. * For 24-h time point for Nascent LNP, a separate mouse from the 16-h time point was shown. The total luminescence counts at 6, 16, 24, 48, and 72 h are quantified and compared between the groups. (**B**) Kinetic curve showing the quantified luminescence units in the flank from each treatment group, with statistics compared between co-tethered and traditional batch transcription systems. All data shown with ± SEM (*n*= 5). Statistical analysis was performed using one-way ANOVA followed by Tukey’s post-test.

While the innate immune infiltration component of the Matrigel study yielded modest differences, the co-tethered system exhibited far superior mRNA expression and luminescence activity compared to the traditional batch transcription system. As shown in the representative IVIS images in Fig. 9A, LNPs containing co-tethered-synthesized NLuc mRNA induced a brighter initial signal compared to the LNPs with traditional batch-synthesized NLuc mRNA, and this signal persisted for longer, resulting in a higher luminescence throughout and at the final time point. After quantifying the luminescence counts using the Perkin Elmer Living Image software, we exhibited consistently improved mRNA expression levels with the co-tethered system. Compared to the traditional batch synthesis system, the LNPs containing co-tethered synthesized NLuc mRNA exhibited 2-fold higher luminescence at 6 h (*P* < .01, Supplementary Fig. S7A), 3-fold higher luminescence at 24 h (Supplementary Fig. S7C), nearly five-fold higher luminescence at 48 h (*P* < .01, Supplementary Fig. S7D), and 1.7-fold higher luminescence at 72 h (Supplementary Fig. S7E). Further, when comparing measurements surrounding the subcutaneous Matrigel to the bioluminescence of nascent LNP alone,

LNPs containing co-tethered-synthesized NLuc mRNA exhibited statistically significant increases at 6 h (*P* < .01, Supplementary Fig. S7A), 48 h (*P* < .001, Supplementary Fig. S7D), and 72 h (*P* < .05, Supplementary Fig. S7E), whereas the LNPs with traditional batch-synthesized NLuc mRNA, while expectedly higher on average, did not exhibit any statistical difference from nascent LNPs at those time points. The kinetic curve of bioluminescent activity in Fig. 9B further conveys this finding, exhibiting the strikingly increased initial bioluminescence indicative of co-tethered NLuc mRNA transfection, which is sustained especially in the later time points between 24 and 72 h. Taken together, these results correlate with and corroborate our previous findings, specifically in conveying that co-tethered transcribed mRNA exhibits improved transfection efficiency compared to traditional batch synthesis methods, as we showed in our RAW264.7 macrophage transfection experiments (Figs 5A and 6). This leads to the explanation that the co-tethered system significantly alters the immunogenicity profile in the transfected cells, allowing stronger and more stable mRNA transfection. Although this reduced immunogenicity may not induce strong differences in innate immune infiltration at the systemic level, it would certainly induce changes at the cellular level, affecting the translational capability of the cells, especially for sensitive immune cells. For example, it is well known that dsRNA induces type-I IFN production, which can induce the activation of protein kinase R and phosphorylation of eIF-2α, therefore halting translation initiation. Since we have confirmed that mRNA produced by the co-tethered system induces statistically lower IFN-β1 expression levels (Figs 5C and D, and 7A), this is likely one of the key mechanisms allowing increased translation for mRNA produced from the co-tethered system relative to its traditional counterpart [47, 48].

## Discussion

Large-scale therapeutic mRNA manufacturing is a multi-step process. Currently, the most involved step is the downstream purification strategy (cellulose-based affinity, RP-HPLC, oligo-dT affinity, or similar purifications) to reduce dsRNA impurities [34, 49–53], as dsRNA contaminants trigger innate immune responses that lead to inflammation and reduced translation [29, 31, 54, 55]. A limitation of current approaches that rely on downstream purification is that removal of dsRNA is generally incomplete due to the inability to distinguish between the secondary structures of mRNA and the same mRNA containing (relatively) small regions of dsRNA. For example, a study using cellulose affinity removal of dsRNA reported 90% removal with 65% yield [50]. Downstream purification also reduces yields and introduces complications in GMP (good manufacturing practices) production.

We recently reported a general approach aimed at eliminating dsRNA production during RNA synthesis [7, 8, 56]. Covalent tethering of the polymerase near the promoter sequence in the DNA drives promoter binding (allowing better competition with RNA rebinding) and allows the use of increased ionic strength in the transcription reaction. Increased ionic strength, in turn, inhibits the RNA rebinding that is a prerequisite for the production of dsRNA additions to the desired RNA. In this general approach, chemical “handles” (for attaching DNA to the polymerase near the promoter and for simultaneous attachment to a surface) were introduced into the DNA via the (chemically synthesized) primers used for PCR production of the templating DNA. Although the PCR approach works well for many applications, particularly for longer mRNAs, users prefer to use plasmid DNA as a template.

### A new approach to labeling plasmid DNA for co-tethered transcription

As illustrated in Fig. 2, we introduce a more general approach for the incorporation of handles, applicable to plasmid (or any other) DNA. Restriction digestion followed by a one-pot polymerase fill-in reaction is accessible to a broad user base across a wide range of applications. The labeling approach allows incorporating chemical handles asymmetrically on both ends (or on just one end) of the linear plasmid DNA. Specifically, the biotin and alkyl-halide-modified plasmid DNA created by DNA polymerase fill-in allows specific 3′ end labeling of DNA with nearly 95% efficiency, as seen from streptavidin bead binding assays. The 3′ end labeled plasmid DNA shows resistance to DNase digestion as expected from the modification.

In the previous study, we demonstrated the general approach with 1 kb NLuc mRNA transcribed from PCR-generated DNA [7]. Here, we extend this to 5.6 kb and 8.6 kb mRNA, now using plasmid DNA. Note that salt sensitivity in the un-tethered batch reaction increases with increasing mRNA length (Figs 3 and 4). This likely reflects the fact that, as mRNA (and plasmid) length increases, for the same mass, molarity decreases with length. Decreased DNA concentrations would be expected to drive less promoter occupancy, yielding a system more sensitive to salt.

In the previous study, to facilitate a general approach to PCR, an attachment of the alkyl-halide linker to the nontemplate strand DNA was at position −29, relative to the transcription start site, requiring a long PEG linker to ensure promoter binding [7]. In the current work, the attachment site along the DNA is closer to the promoter, at position −21 in the template strand (−18 for the 5.6 kb Cas9–EGFP mRNA), and we have used a much shorter linkage to the alkyl halide. It is likely that further empirical optimization can be achieved, considering both linker length and attachment position along the DNA, which determines both linear distance and the helical rotation of the attachment point (note that DNA end breathing may soften such requirements).

Co-tethering provides the unique advantage that the immobilized polymerase-DNA catalyst can readily be (re-)used for multiple rounds of synthesis. Note that in our previous study with 1 kb NLuc mRNA, we achieved near-constant yields across 20 cycles of transcription. The results in Fig. 4 show similar benefits, with some decrease in yield at high cycle numbers. This is most prominent for the 8.6 kb mRNA, and we suspect this may be the result of trace amounts of DNA endonucleases in the protein or DNA preps. These would minimally impact a single 2 h transcription reaction but could manifest over many hours. Longer RNAs, such as the 8.6 kb mRNA here, encoded by longer DNAs, provide more endonucleolytic targets per RNA, and the DNA is present at a lower molar concentration. This is a potential problem for any transcription reaction, but in this system, any cleaved fragments, containing the polymerase bound to the promoter, will wash away at each cycle, reducing yield, but also limiting the synthesis of mRNAs from the truncated DNAs.

### High stringency, functional assays of mRNA purity

RNA produced by this system is recovered free of enzyme and DNA and so does not require purification to remove these macromolecules (and unlike traditional batch reactions, DNase is not added to remove DNA). We have shown previously that mRNA produced by the co-tethered system at 0.3 M NaCl shows significantly reduced dsRNA production [7]. In earlier work, we showed low innate immunogenicity with the transfection of a similarly synthesized 0.8 kb mRNA into HEK293T cells. In the current work, we have extended this analysis to more sensitive macrophage cells.

For this study, mRNA was synthesized in the functionally co-tethered system at 0.3 M salt (given the analyses in Fig. 3, a lower salt concentration might provide similar quality) and compared to mRNA synthesized in a traditional batch reaction (necessarily at low salt). Transfection of NLuc mRNA into mammalian RAW264.7 macrophage cells, shown in Fig. 6, reveals a dramatic difference between traditional batch-produced mRNA and mRNA produced using this co-tethered approach. Innate immune stimulation, as measured by upregulation of IFN-β1, is decreased about 40-fold, and since innate immune stimulation down-regulates translation, intracellular NLuc expression increases about 60-fold.

Replacement of UTP by m1ψ reduced the immunogenicity of the batch-synthesized mRNA but offered no measurable improvement in high salt, co-tethered transcription. Indeed, co-tethered synthetic mRNA with UTP performs significantly better than batch-synthesized mRNA with m1ψ. Similarly, purification with oligo d(T)_25_ to reduce dsRNA offers only a slight improvement to mRNA performance (this may also arise from removing small amounts of truncated mRNAs, as the assays were done with constant mass of RNA).

For mRNA synthesized by the co-tethered system, both 0.8 kb NLuc and 5.6 kb Cas9–EGFP show increased cellular expression and reduced innate immune activation as measured by type-I interferon activity when transfected into both human epithelial-like cells (e.g. HEK293T) and mouse undifferentiated macrophage cells (e.g. RAW264.7). We have further studied the upregulation of the cytosolic receptor RIG-I that can bind to the triphosphorylated 5′ terminal of RNA duplexes and bind to shorter dsRNA fragments. We observed upregulation of RIG-I receptors compared to untreated cells with traditional batch-synthesized mRNA, whereas the upregulation was minimal with mRNA produced by the co-tethered system. This suggests that traditional batch synthesis of mRNA produces shorter dsRNA residues, which, when not extensively purified, can trigger RIG-I-mediated pro-inflammatory responses [57]. All the mRNA used for these studies is treated with Antarctic phosphatase to remove the 5′ triphosphate from uncapped mRNA, which does not contribute towards RIG-I induction.

This study validates the *in vivo* performance of NLuc mRNA produced by the co-tethered transcription system using a novel subcutaneous Matrigel plug mouse model. Mice were injected with Matrigel mixed with LNPs containing mRNA made via co-tethered or traditional batch synthesis methods, or empty LNPs. Bioluminescence imaging tracked mRNA expression over 72 h, followed by immune profiling of the Matrigel to assess immune cell infiltration. This model enabled simultaneous evaluation of both immunogenicity and mRNA expression, demonstrating the system’s translational potential. The study found that while co-tethered transcription reduced active DCs and T cells, the primary driver of immune cell recruitment in the Matrigel model was the ionizable lipid nanocarriers themselves. This aligns with prior research showing that ionizable lipids, such as those used in COVID-19 vaccines, can trigger strong immune responses, including neutrophil infiltration and activation of inflammatory pathways like NF-κB and NLRP3. These effects explain the high immune cell presence even with empty LNPs and why mRNA transcription had little additional impact on systemic immune recruitment.

## Conclusions

This study demonstrates a robust and facile approach for preparing a functionally tethered co-catalyst comprised of T7 RNA polymerase covalently attached to a DNA template encoding RNA (of any length) and immobilized on a solid support. Engineered restriction sites, outside of the final encoding region, allow for a one-pot labeling using standard DNA polymerase fill-in biochemistry. Immobilization on magnetic beads ensures that all RNA polymerase is covalently partnered with DNA and allows for facile re-use of both for multiple rounds of transcription. The utility is demonstrated with plasmids encoding 0.8, 5.6, and 8.6 kb mRNA targets. As demonstrated previously, transcription is resistant to elevated NaCl concentrations that inhibit product RNA re-binding to RNA polymerase. Elimination of dsRNA contamination in the initial product yields mRNA that expresses well, with very low innate immune stimulation (with or without *N*^1^-methyl pseudouridine substituting for uridine). In standard HEK293T cells, expression (of both 0.8 kb NLuc mRNA and 5.6 kb Cas9–EGFP mRNA) shows a dramatic improvement over traditional batch synthesis. Purity is further illustrated by high differential expression in more sensitive RAW264.7 macrophage cells, with correspondingly low innate immune stimulation. Finally, results in a new subcutaneous Matrigel mouse model are presented, and again, RNA produced in a functionally co-tethered, salt-challenged reaction performs better than RNA produced in a traditional batch reaction. These results pave the way for a simplified path to mRNA of exceptional purity, requiring essentially no purification post-synthesis.

## Supplementary Material

gkaf1355_Supplemental_Files

## Data Availability

Data available on request and at https://osf.io/enrp6/.
