# Supplementary material for: A novel plasmid-based co-tethered transcription platform for high yield, high purity mRNA synthesis
Source: Nucleic Acids Res. 2025 Dec 29;53(22):gkaf1355. doi: 10.1093/nar/gkaf1355 (PMC12746099; doi:10.1093/nar/gkaf1355)
Supplement: gkaf1355_Supplemental_Files [file gkaf1355_supplemental_files.zip › Supplementary Material NAR-02547-Met-K-2025 Revised.pdf]

A Novel Plasmid-Based Co-Tethered Transcription Platform for High Yield, High Purity mRNA Synthesis

Purnima Mala[1†], Ruptanu Banerjee[1†], Amin Abek[1], James Forster III[2], Aniruddha Pinjari[2], Ashish A. Kulkarni[2], and Craig T. Martin[1]*
Department of Chemistry[1]
Department of Chemical Engineering[2]
University of Massachusetts Amherst, USA
[†]Contributed equally

SUPPLEMENTARY MATERIAL

Table of contents:

Figure S1. Synthesis and Incorporation of Cl-alkyl-dUTP into DNA via Klenow Fill-In Reaction 2

Figure S2. Streptavidin Bead Binding Efficiency for Biotinylated DNA Templates 2

Figure S3. Stability of co-tethered enzyme-DNA immobilized complex 3

Figure S4. Run-off transcription analysis of 70 base RNA in 20% 7M urea PAGE 4

Figure S5. NLuc-LNP formulation and characterization 4

Figure S6-S8. *In vivo* Matrigel plug model to assess mRNA expression and immune cell invasion 5

Supplementary Table I. DNA primer sequences used in RT-qPCR 6

Plasmid sequence data available as GeneBank files:

NLuc 0.8.gb	Encodes nanoluciferase
Cas9-EGFP 5.6.gb	Encodes Cas9-EGFP fusion
Emerin-EGFP 8.6.gb	Encodes Emerin-EGFP fusion

Synthesis and Incorporation of Cl-alkyl-dUTP into DNA via Klenow Fill-In Reaction
The modified nucleotide Cl-alkyl-dUTP is not currently available commercially. To synthesize this reagent, HaloTag® succinimidyl ester (O4) was reacted with amino-allyl-dUTP in PBS buffer (pH 7.4) at room temperature, as outlined in Figure 2B. This in-house approach provides greater flexibility in DNA labeling compared to post-synthetic modification strategies. Notably, the Klenow fragment DNA polymerase efficiently incorporated Cl-alkyl-dUTP during a fill-in reaction at a 3′ recessed DNA end. The Klenow-mediated fill-in reaction on an annealed double-stranded DNA template resulted in near-complete incorporation (~100% efficiency), as shown in Figure 2B, lane 4. Both unmodified and modified DNA products were analyzed using 20% denaturing PAGE with 7 M urea (Supplementary Figure S1, lanes 3 and 4).

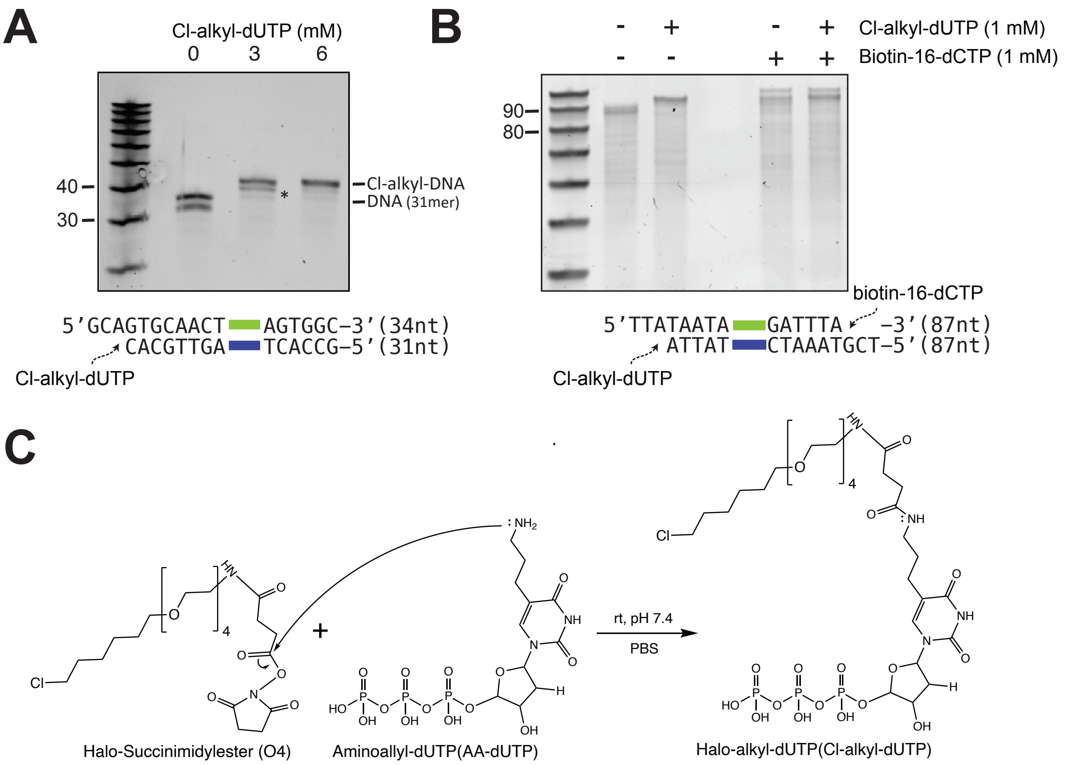

Figure S1. (A) A Klenow fill-in reaction was performed on a 31 bp double-stranded DNA substrate to evaluate the proof of concept for the simultaneous incorporation of aminoallyl-dUTP and a Halo ligand. Complete incorporation was observed, as indicated by the band in lane 4. (B) Klenow fill-in reaction demonstrating incorporation of biotin-dCTP and aminoallyl-dUTP extended into 87 bp dsDNA. Distinct band shifts in lanes 3, 4, and 5 relative to the control (lane 2) confirm the successful incorporation of modified nucleotides. (C) Schematic representation of aminoallyl-dUTP synthesis. A test "Klenow fill-in" reaction with annealed dsDNA template shows ~95% incorporation efficiency of Cl-alkyl-dUTP.

Streptavidin Bead Binding Efficiency for Biotinylated DNA Templates

Biotin-labeled Nanoluc DNA incubated with streptavidin-coated beads demonstrated efficient and specific binding The binding capacity of biotin-labeled plasmid DNA templates was evaluated using streptavidin-coated magnetic beads. Biotinylation was achieved via a Klenow fragment-mediated fill-in reaction incorporating biotin-16-dCTP, yielding an incorporation efficiency exceeding ~95%. When the 806 bp NLuc-encoding DNA template was incubated with hydrophilic magnetic streptavidin beads, complete binding of the biotinylated DNA was observed (Figure S2, lane 2). Similarly, the 8647 bp ELYS-Emerin-EGFP-encoding DNA template demonstrated ~95% binding efficiency following biotinylation, as indicated by the upper band in Figure S2, lane 5.

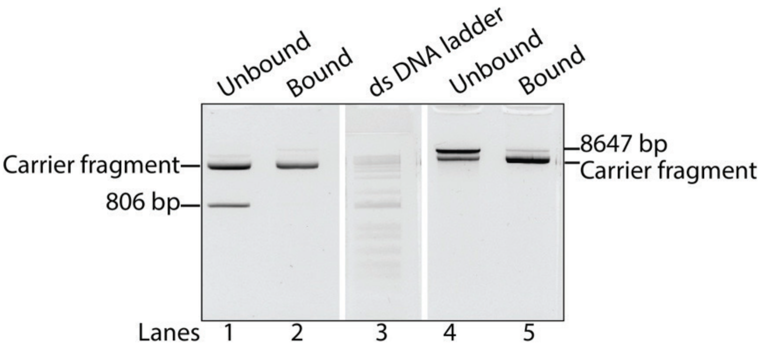

Figure S2. Evaluation of DNA Binding Efficiency to Streptavidin-Coated Beads via Klenow-Based Biotin Labeling in 2% agarose gel.

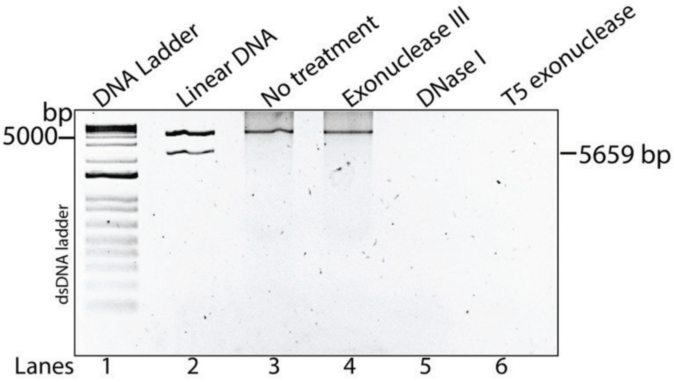

Figure S3. Stability of co-tethered enzyme-DNA immobilized complex. A 2% agarose gel electrophoresis was performed to assess the stability of co-tethered enzyme–DNA complexes in the presence of various DNases. In Lane 2, two distinct bands are observed following BspQ1 digestion of a 5.6 kb RNA-encoding plasmid, with the upper band corresponding to the template DNA modified at the 3' end. Lane 3 contains an intact DNA band, representing the untreated co-tethered enzyme–DNA complex and serving as a positive control. In Lane 4, a retained DNA band indicates resistance to Exonuclease III (3'→5' activity), attributable to the 3' end modification of the template DNA. Lanes 5 and 6 show a complete absence of DNA bands, consistent with degradation by exonucleases due to the lack of protective 5' end modifications.

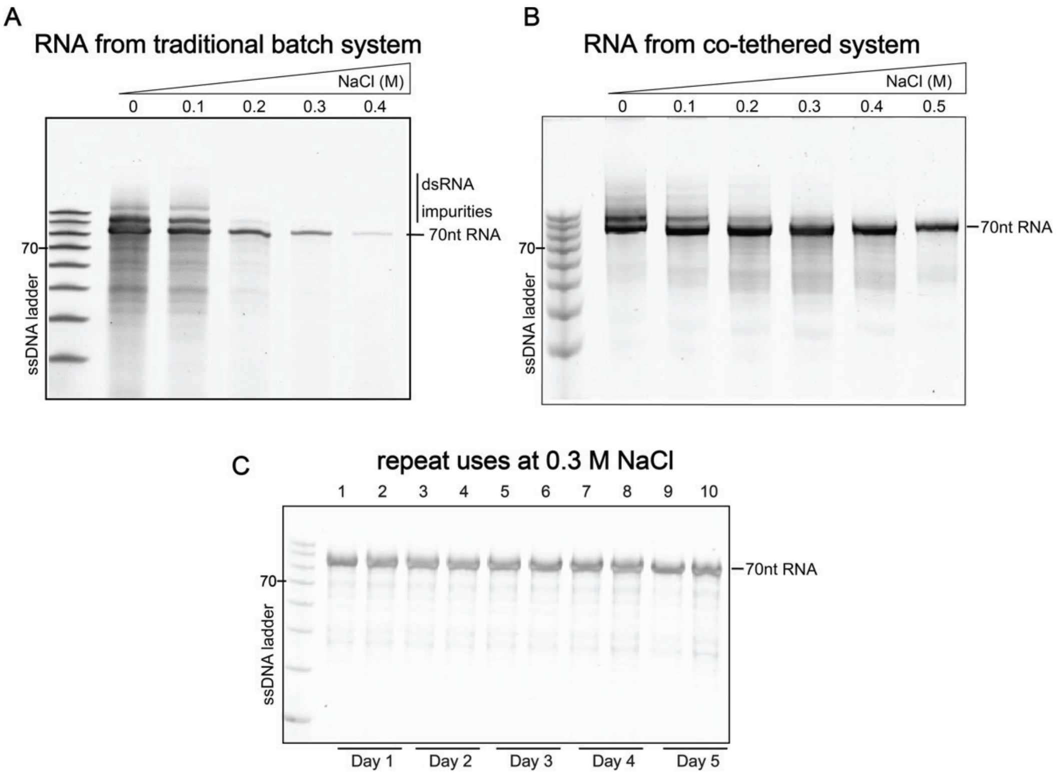

Figure S4. Run-off transcription analysis of 70 base RNA in 20% 7M urea PAGE. A) Free DNA-polymerase generates dsRNA impurities and decreased yield of RNA at higher salt. B) Co-tethered DNA-polymerase tolerates higher salt and generates less dsRNA impurities. C) Repeat-batch transcription at 0.3M salt from the same co-tethered complex. Salt-resistant synthesis of 0.8 to 8.6 kb mRNAs.

NLuc-LNP formulation and characterization

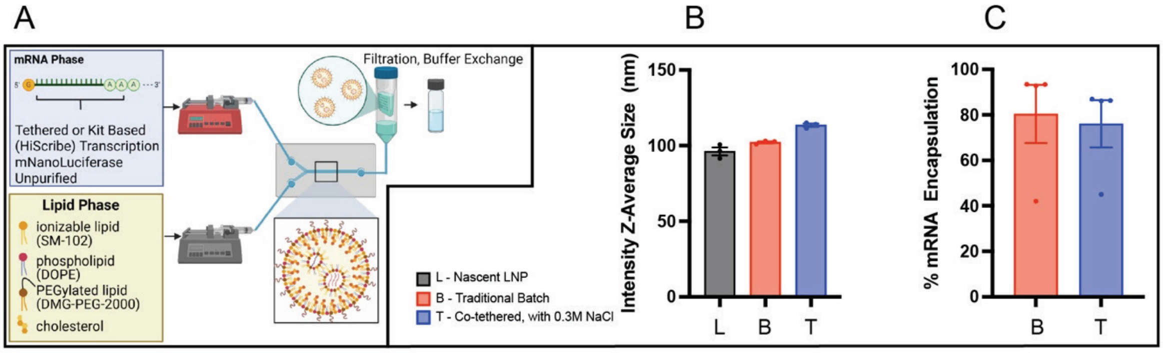

Figure S5. Assessment of co-tethered transcribed NLuc mRNA encapsulated in lipid nanoparticles (LNPs) for delivery in an immune cell infiltration Matrigel plug mouse model (**A**) Schematic showing microfluidic assembly workflow for the mRNA-LNPs. (**B**) Intensity average size and (**C**) encapsulation efficiency of the LNPs. Data shown is ± SEM (n=3).

In vivo Matrigel plug model to assess mRNA expression and immune cell invasion

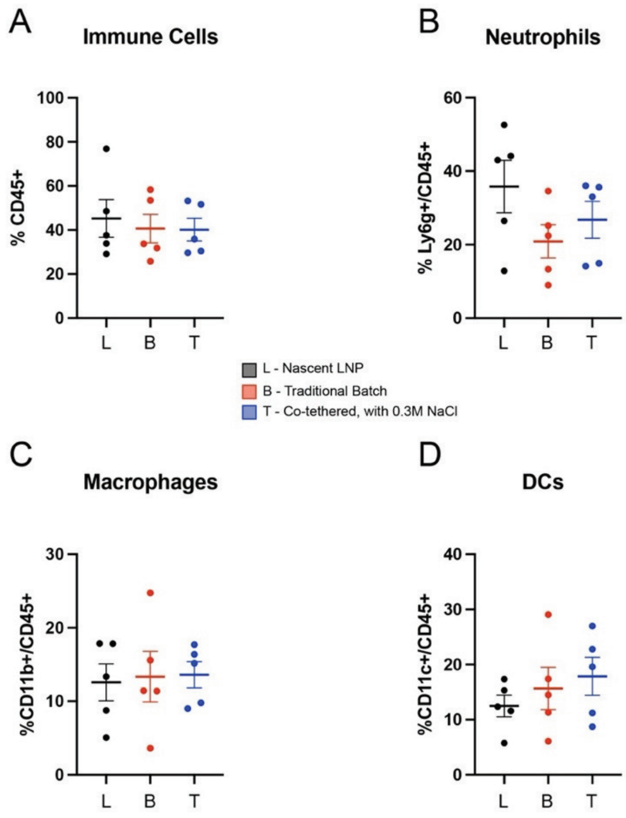

Figure S6: Percentage of (A) CD45.2+ immune cells, (B) Ly6G+ neutrophils, (C) total F4/80+ macrophages, and (D) total CD11c+ dendritic cells infiltrated into the Matrigel

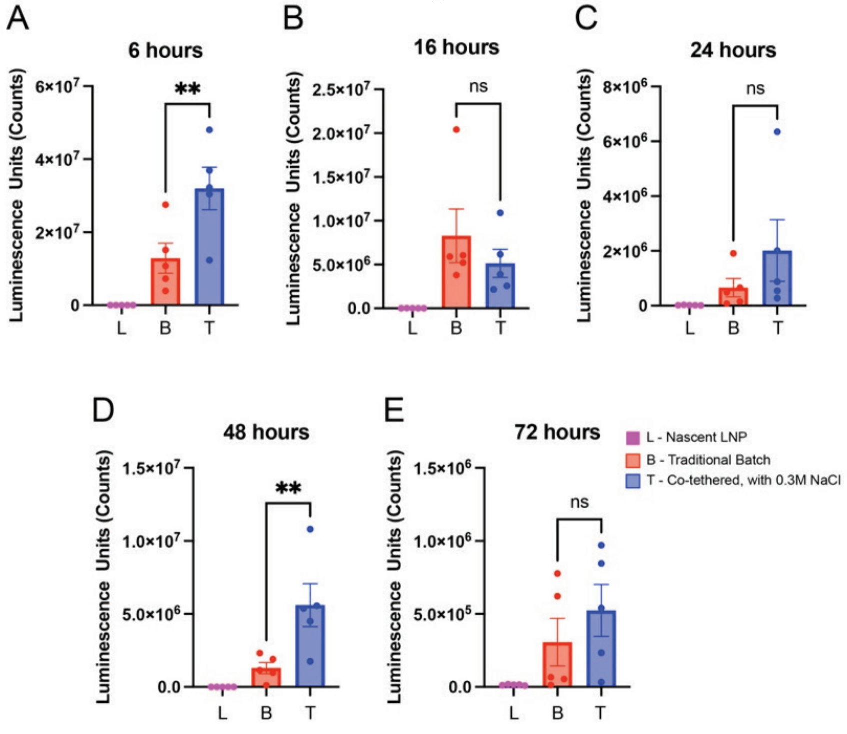

Figure S7. Total luminescence counts at (A) 6, (B)16, (C) 24, (D) 48, and (E) 72 hours time-points from IVIS. Corresponding images taken using IVIS are in Figure 9A.

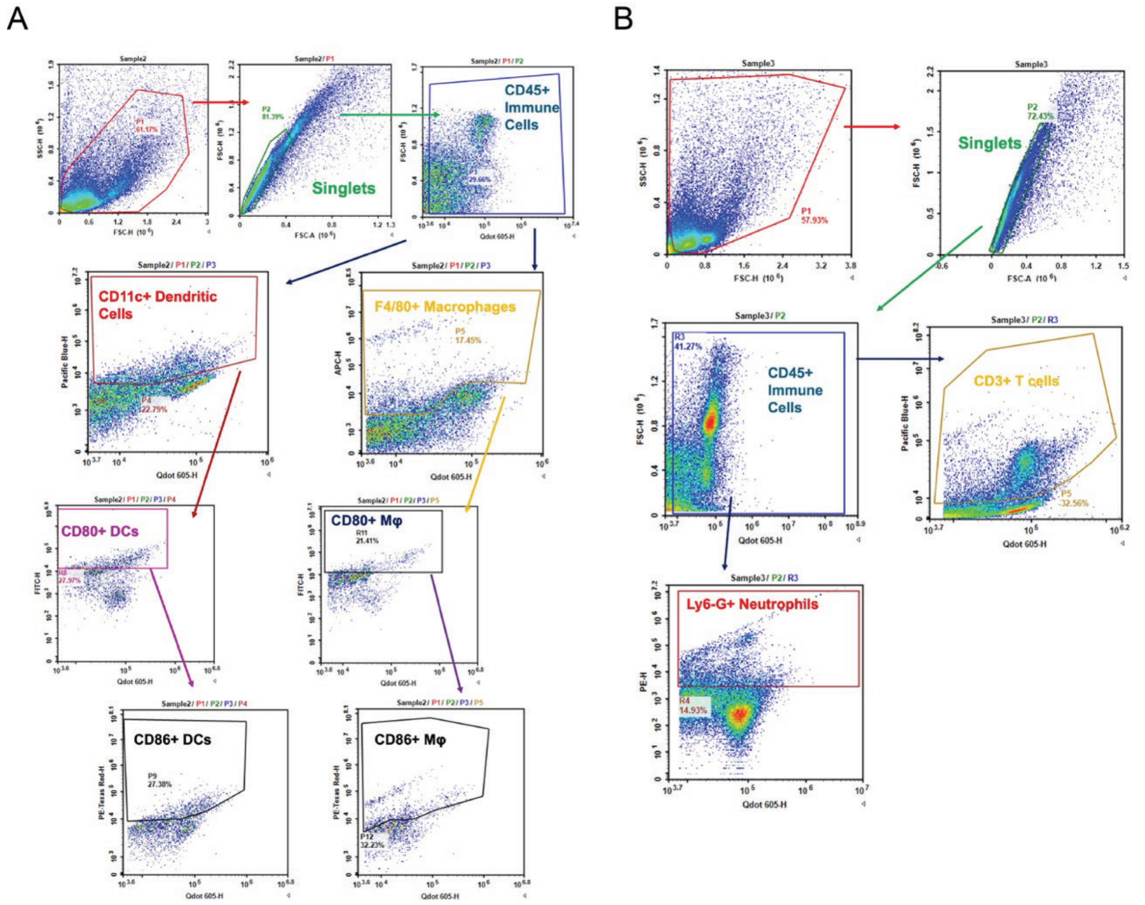

Figure S8. Representative gating strategy for (A) Panel A (macrophages, dendritic cells and co-stimulatory receptors) and (B) Panel B (T cells and neutrophils) from the ex vivo Matrigel flow cytometry

Supplementary Table I: DNA primer sequences used in RT-qPCR (Figure 6 B, C &D)

Gene	Direction	Sequence (5'→ 3')
Human GAPDH	Forward	ATT CCA CCC ATG GCA AAT TC
	Reverse	TGG GAT TTC CAT TGA TGA CAA G
Human IFNB1	Forward	TTC AGT GTC AGA AGC TCC TGT GG
	Reverse	CTG CTT AAT CTC CTC AGG GAT GTC A
Human RIG-I (DDX58)	Forward	TGG ACC CTA CCT ACA TCC TG
	Reverse	TCA GCC TGA ATA TAC TGC AC
Mouse GAPDH	Forward	TCT TGG GCT ACA CTG AGG AC
	Reverse	CAT ACC AGG AAA TGA GCT TGA
Mouse IFNB1	Forward	CCC TAT GGA GAT GAC GGA GA
	Reverse	CTG TCT GCT GGT GGA GTT CA
Mouse RIG-I (DDX58)	Forward	CTG CCT CAC TCT TCC TCC AG
	Reverse	TGG CTT CAC AAA GTC CAC AG
